# Temporal dynamics outweigh spatial gradients in shaping water quality across a regulated Afrotropical reservoir cascade

**DOI:** 10.1007/s10661-026-15681-8

**Published:** 2026-08-03

**Authors:** Abdullahi Ibrahim, Suleiman Omeiza Eku Sadiku, Deborah Vivienne Robertson-Andersson, Moses Okpeku

**Affiliations:** 1 Discipline of Biology, School of Agriculture and Sciences, University of KwaZulu-Natal, Westville Campus, Durban, South Africa; 2 Department of Water Resources, Aquaculture and Fisheries Technology, School of Food Science and Agricultural Technology, Federal University of Technology, Minna, Nigeria; 3 Chrysalis Training and Skills Development, Kei Mouth, South Africa

**Keywords:** Reservoir water quality, Temporal variability, Sentinel parameters, Land-use impacts, Niger River Basin, Tropical Africa

## Abstract

Water quality in regulated tropical reservoirs is shaped by the interplay of seasonal hydrology, spatial habitat heterogeneity, land use, and climate variability, yet the relative importance of these drivers remains poorly quantified in African river basins. We aimed to partition the variance in water quality among temporal, spatial, land-use, and climate drivers across the Kainji–Jebba reservoir cascade in Nigeria and to identify sentinel parameters capable of consistently and timely detecting water-quality change. Monthly water-quality surveys were conducted at six sites spanning two dams and three habitat types (riverine, ecotonal, and lacustrine) from April 2024 to March 2025 (*n* = 72 samples). A modified National Sanitation Foundation Water Quality Index (WQI) was calculated for each sample. Variance partitioning using partial redundancy analysis (RDA), exploratory Granger causality testing, and six complementary changepoint detection methods were applied to disentangle the relative contributions of space, time, land use, and climate. Temporal variation (season and month) explained 28.3% of the variance in water quality, whereas spatial factors accounted for 1.8% and land-use effects for 0.4%. These results suggest that seasonal hydrological dynamics were the dominant structuring force within the study period, although the limited spatial extent of sampling (six sites across two reservoirs) should be considered when interpreting the comparatively weak spatial signal. After Benjamini–Hochberg false-discovery-rate correction, no climate–water-quality relationships were statistically significant across 480 stratified Granger causality tests; given the exploratory scope of the analysis and the 1-year dataset, this null result is treated as hypothesis-generating rather than conclusive. WQI, electrical conductivity (EC), and relative humidity (RH) showed the highest changepoint consensus detection rates (71.4% each) and are proposed as sentinel parameters for water-quality monitoring, although the role of RH warrants further investigation before operational adoption. These findings suggest that monitoring programmes in regulated tropical reservoir cascades may benefit from prioritising targeted seasonal windows, the wet-season runoff peak (June–July) and the black flood period (January–February), rather than expanding spatial coverage, pending validation across longer time series. The integrated analytical framework, combining WQI, variance partitioning, exploratory Granger causality, and multi-method changepoint detection, offers a transferable template for other data-poor tropical river systems where monitoring resources must be allocated efficiently.

**Supplementary information:**

The online version contains supplementary material available at https://doi.org/10.1007/s10661-026-15681-8.

## Introduction

Freshwater ecosystems underpin drinking water supply, inland fisheries, agriculture, and regional climate regulation, yet they are among the most rapidly degrading ecosystems on Earth. Although they cover less than 1% of Earth’s surface, they support more than 10% of all animal species and over half of all known fish species (Reid et al., 2019; Albert et al., 2021). Freshwater vertebrate populations have declined by 84% since 1970, and nearly one-third of freshwater fish species are now threatened with extinction (Tickner et al., 2020; WWF, 2020). Dams are a principal driver: approximately 58,000 large dams now fragment 60% of the world’s rivers (Grill et al., 2019; Barbarossa et al., 2020). The principal mechanisms by which dams alter river ecosystems are well established: dams retain sediment and nutrients (Søndergaard et al., 1992), homogenise downstream flow regimes, replace fast-flowing oxygenated river water with slower and often thermally stratified reservoir water, and sever lateral floodplain connections that sustain aquatic food webs (Ward & Stanford, 1995; Poff et al., 1997; Tockner & Stanford, 2002). In temperate rivers, the River Continuum Concept predicts orderly downstream ecological gradients, with dams introducing discrete discontinuities along that continuum (Vannote et al., 1980). In tropical rivers, however, seasonal flood pulses, rather than longitudinal position, are often the dominant organising force: annual inundation reconnects channels and floodplains, mobilises nutrients stored in terrestrial soils, and synchronises fish reproduction in ways that flow regulation can dampen or disrupt entirely (Junk et al., 1989; Wantzen et al., 2008). Whether regulated tropical rivers retain sufficient hydrological connectivity—through spill events, seasonal drawdown, and fish movement—to buffer these effects remains incompletely resolved (Walsh et al., 2022; O’Mara et al., 2024). Catchment-scale land-use change adds further pressure: deforestation weakens riparian buffering and accelerates runoff delivery of nutrients and sediments, while rapid urban growth in areas with inadequate sanitation infrastructure increases the direct discharge of untreated domestic and industrial effluent into river networks, collectively contributing to the near-doubling of global riverine nitrogen transport observed over recent decades (Beusen et al., 2016; Jones et al., 2021; Oester et al., 2025). Yet relatively few studies have simultaneously quantified the relative contributions of hydrology, space, land use, and climate to water-quality variation in tropical African basins, precisely the systems in which cost-effective monitoring is most urgently needed (Faye et al., 2023; Yates et al., 2025).

Sub-Saharan Africa illustrates both the ecological importance of this problem and the inadequacy of the evidence base available to address it. The region supports exceptional freshwater biodiversity and high basin-level endemism, yet research output remains sparse and geographically concentrated (Pototsky & Cresswell, 2021; Chapman et al., 2022). Nigeria alone harbours more than 300 freshwater fish species; FishBase currently lists 334 species for the country, of which 311 are recorded as presently occurring (Froese & Pauly, 2025). Yet systematic water-quality research in the country remains limited. This evidence gap is especially consequential in the Upper Niger Basin, where the Kainji and Jebba dams, commissioned in 1968 and 1984, respectively, have regulated one of West Africa’s most ecologically and socially significant river systems for over four decades, yet little evidence exists of any published basin-wide water-quality assessment spanning the cascade (Oyebande, 2001; Oyebande & Odunuga, 2010). The human cost has been measurable and severe: following impoundment, artisanal fishery catches collapsed from roughly 28,000 to approximately 8000 tonnes per year, stripping riparian communities of their primary protein source and principal livelihood, while reduced downstream sediment delivery degraded the floodplain soils that previously renewed agricultural fertility during seasonal inundation (Ita et al., 1985; Arthington et al., 2006; Olaosebikan & Raji, 2013; Arantes et al., 2019; FAO, 2020). These dam-mediated changes have since been compounded by accelerating catchment degradation: satellite-derived land-cover analyses indicate that agriculture now occupies a substantial proportion of reservoir catchment area and that riparian forest loss and urban expansion are ongoing, increasing runoff-borne nutrient and sediment loads while progressively weakening natural buffering capacity (Lanrewaju, 2012; Hansen et al., 2013; Zanaga et al., 2022; Eteh et al., 2024). Dense water-hyacinth infestations along reservoir margins intensify oxygen depletion by elevating biochemical oxygen demand during decomposition and suppressing wind-driven surface reaeration, thereby generating hypoxic conditions harmful to aquatic biota (Pelikán & Marková, 2013; Uwadiae et al., 2021). Climate projections for West Africa further indicate increasing hydroclimatic variability and continued warming by mid-century, with likely consequences for wet-season pollutant mobilisation and dry-season concentration of dissolved constituents (Sylla et al., 2018; IPCC, 2022). Resolving how these interacting stressors partition their influence on water quality is therefore both an ecological and a public-health imperative (Mbaka & Mwaniki, 2017; Jézéquel et al., 2020).

Recent advances in remote sensing and statistical ecology make rigorous driver partitioning feasible even in data-limited systems. Partial redundancy analysis (RDA) with variance partitioning separates the independent and shared contributions of spatial, temporal, land-use, and climate predictors to multivariate water-quality variation (Borcard et al., 2018). Granger causality analysis provides an exploratory test of whether climate variables contain predictive information about future water-quality states beyond what the water-quality series itself can predict (Zolghadr-Asli et al., 2021). Multi-method changepoint detection identifies abrupt transitions between water-quality states and supports the selection of parameters that respond early and consistently across analytical frameworks (Scheffer et al., 2009; Lund et al., 2023). The National Sanitation Foundation Water Quality Index (WQI) translates multivariate physicochemical observations into a single management-relevant score accessible to water-resource managers and regulators (Aljanabi et al., 2021; Uddin et al., 2021; Chidiac et al., 2023). We applied this integrated framework to monthly water-quality surveys conducted from April 2024 through March 2025 across six sites spanning the Kainji–Jebba reservoir cascade, covering one complete hydrological cycle. Three research questions structured the analysis:

Do the Kainji and Jebba dams create distinct spatial water-quality zones consistent with temperate river theory, or does hydrological connectivity maintain basin-wide temporal coherence more consistent with the flood pulse concept?

What explains temporal variation in water quality—seasonal hydrology, climate variability, or catchment land use acting through runoff pulses—and what is the relative contribution of each driver?

Which water-quality parameters detect change earliest and most reliably, and can a parsimonious set of sentinel parameters support cost-effective monitoring across this cascade?

These questions extend beyond Nigeria. Across tropical Africa, Asia, and Latin America, many regulated river systems face analogous combinations of dam regulation, land-use change, and climate pressure under severe monitoring resource constraints. In data-poor regulated rivers, the key management issue is not how to monitor everywhere, but when and where to allocate monitoring effort to yield the greatest return. A transferable analytical framework for answering that question could meaningfully improve the efficiency of conservation investment across tropical freshwater systems (Yates et al., 2025).

## Materials and methods

### Study area

**Fig. 1:**
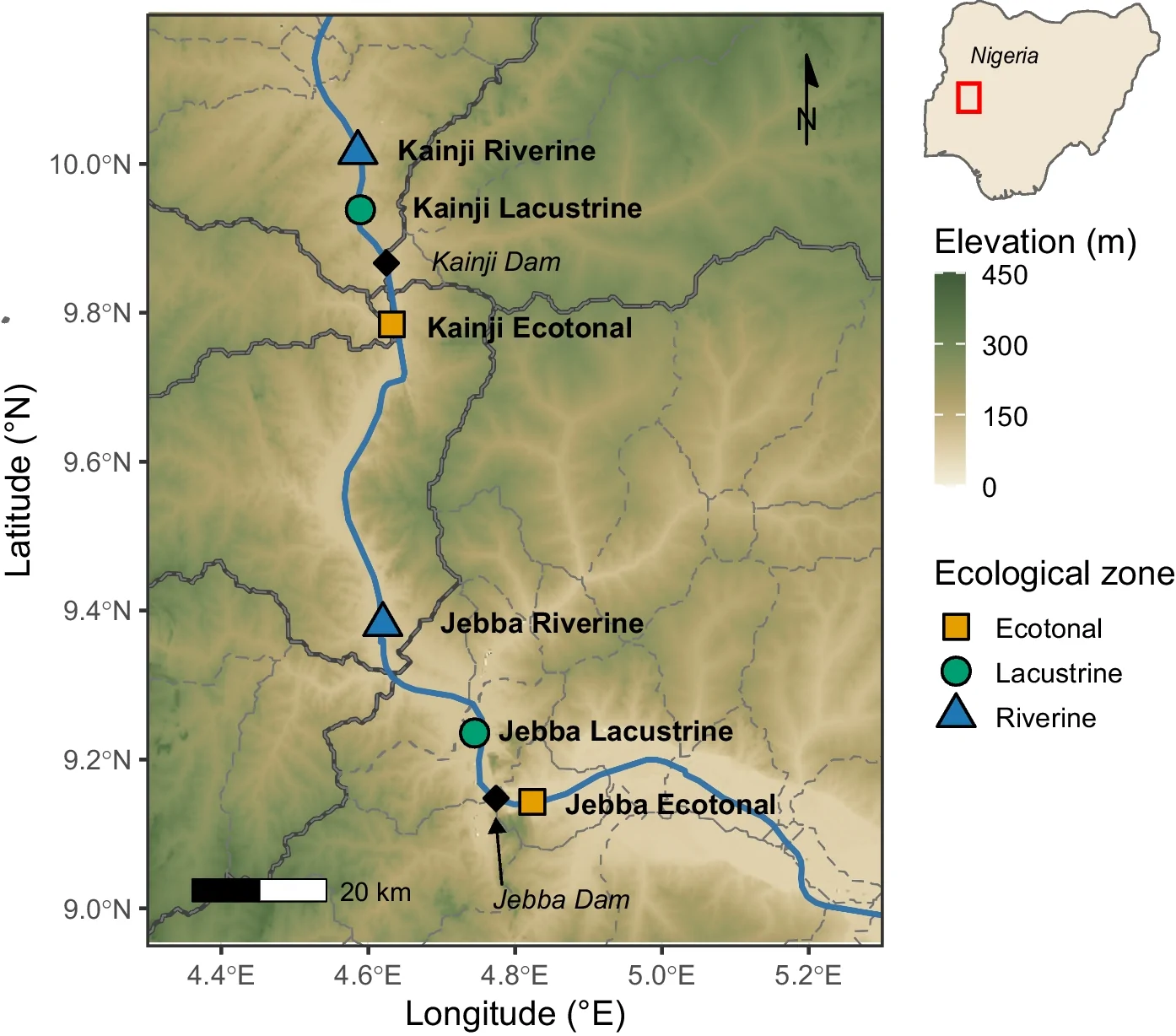
Study area and sampling sites at Kainji and Jebba dams along the Upper Niger River, Nigeria. Sites: Kainji Dam Riverine-Upstream: Garafini, Kainji Dam Ecotonal-Transition: Awuru, Kainji Dam Lacustrine-Reservoir: Yuna, Jebba Dam Riverine-Upstream: Gbajibo, Jebba Dam Ecotonal-Transition: Juju Rock, Jebba Dam Lacustrine-Reservoir: Kokodi. Monthly sampling conducted April 2024–March 2025 (*n* = 72 samples). Coordinate system: WGS 84

The study was conducted in Nigeria’s Upper Niger River Basin, centred on the Kainji–Jebba reservoir cascade (Fig. 1). Kainji Dam (10.5° N, 4.6° E; commissioned 1968) and Jebba Dam (9.1° N, 4.8° E; commissioned 1985) form a sequential hydroelectric cascade along the Niger River with a combined installed capacity of approximately 1338 MW (Chiromo et al., 2016). The region experiences a tropical savanna climate with a pronounced wet season (April–October) and dry season (November–March), receiving mean annual rainfall of 1100–1400 mm (Adegbehin et al., 2016). These seasonal transitions generate hydrological pulses that structure aquatic productivity across the cascade.

At each dam, we sampled three habitat types that collectively capture the full environmental gradient imposed by impoundment: riverine zones (upstream free‑flowing sections; current velocity 0.3–0.8 m s^−1^), ecotonal zones (transitional areas between flowing river sections and reservoir backwaters, where mixing of lotic and lentic water masses creates chemically dynamic conditions), and lacustrine zones (deep reservoir cores, > 8 m depth, with negligible current and potential for thermal stratification; Wetzel, 2001). The six sampling sites were as follows: Kainji Riverine (Garafini: 10.01555° N, 4.58584° E), Kainji Ecotonal (Awuru: 9.78447° N, 4.63231° E), Kainji Lacustrine (Yuna: 9.92093° N, 4.58925° E), Jebba Riverine (Gbajibo: 9.38147° N, 4.61988° E), Jebba Ecotonal (Juju Rock: 9.14158° N, 4.80761° E), and Jebba Lacustrine (Kokodi: 9.20615° N, 4.75372° E). Sites were georeferenced using a Garmin eTrex 30 × GPS and mapped using the sf package in R 4.5.1 (Bivand et al., 2013).

### Study design and temporal scope

We adopted a nested sampling design, sites nested within habitat types, which were in turn nested within dams, with monthly surveys conducted from April 2024 through March 2025, yielding 12 temporal replicates at each of six sites (*n* = 72 total samples). This hierarchical structure follows established protocols for partitioning spatial and temporal variance in lotic systems (Underwood, 1997). The design captures one complete hydrological cycle while remaining logistically feasible for a resource‑constrained field programme. Each site was sampled once per month between 06:00 and 10:00 h to minimise diel variation in dissolved oxygen and temperature. All six sites were sampled within 5–7 consecutive days each month to reduce temporal confounding when assessing spatial patterns.

### Water quality sampling and laboratory analysis

Water samples were transported to the National Institute for Freshwater Fisheries Research (NIFFR) Water Quality Laboratory within 6 h of collection and analysed in duplicate; coefficients of variation ranged from 2.1% to 8.7%, indicating acceptable analytical precision. Surface water (0.5 m depth) was collected using a Van Dorn horizontal water sampler (Wildco, 1.2 L) at lacustrine sites accessed by motorised boat and by grab sampling at riverine and ecotonal sites. Samples were stored in acid‑washed polyethene bottles at 4–6 °C during transport.

Water temperature (*T*~w~), pH, and electrical conductivity (EC) were measured in situ using a calibrated multi‑parameter probe (Bluelab Combo Meter), while dissolved oxygen was measured using a portable metre (Milwaukee MW600 PRO). Water clarity was recorded as Secchi disc depth (m) and converted to a relative turbidity proxy (SD^−1^) for index scoring following Davies‑Colley and Smith (2001); these derived values are treated as comparative proxies rather than instrument‑measured nephelometric turbidity units. Nitrate‑nitrogen (NO₃‑N) and phosphate‑phosphorus (PO₄‑P) were determined by UV–Vis spectrophotometry following APHA standard colourimetric procedures (APHA, 2017). Biochemical oxygen demand (BOD₅) was determined by 5‑day incubation at 20 °C.

### Meteorological data and land-use characterisation

Monthly meteorological data: air temperature (T~a~), rainfall, relative humidity, wind speed, and atmospheric pressure, corresponding to the 12‑month study period (April 2024–March 2025) were obtained from stations operated by the National Institute for Freshwater Fisheries Research (NIFFR) at New Bussa (for Kainji sites) and by Mainstream Energy Solutions at Jebba (for Jebba sites), with both stations validated by the Nigerian Meteorological Agency. No multi‑year climate record was available for these validated stations; accordingly, all Granger causality analyses are treated as an exploratory screening exercise within a single‑year time frame rather than as a confirmatory test of long‑term climatic forcing.

Land‑cover composition was extracted from ESA WorldCover 2021 (10‑m resolution; Zanaga et al., 2022) using 5‑km radius buffers around each sampling point. This radius approximates the hydrological contributing area influencing water quality at monthly sampling intervals under typical dry‑season flow velocities in the Upper Niger and is consistent with buffer scales applied in previous tropical reservoir catchment studies (Carvalho et al., 2019). The percentage cover of agricultural, urban, and forest land was calculated for each buffer. Moran’s *I* indicated moderate spatial autocorrelation in agricultural cover (*I* = 0.42, *p* = 0.031) but not in urban or forest cover. Buffer‑based land‑cover metrics provide a reproducible first‑order approximation of catchment conditions but do not fully capture hydrological connectivity, preferential flow pathways, or pollutant transport processes; land‑use results are interpreted accordingly.

### Water Quality Index (WQI) calculation

We calculated WQI using a modified National Sanitation Foundation method (Brown et al., 1970), aggregating eight parameters: dissolved oxygen, pH, BOD₅, T~w~, NO₃-N, PO₄-P, turbidity, and electrical conductivity. Faecal coliforms were excluded because they primarily reflect public health risk rather than ecological integrity for fish. Each parameter was assigned a rescaled weight summing to 1.0 after redistributing the excluded faecal coliform weight (Table 1). Raw measurements were converted to dimensionless sub-indices (Qᵢ; 0–100) using NSF-based piecewise-linear transformation functions (Brown et al., 1970), and the composite WQI was calculated as WQI = Σ(*Q*ᵢ × *W*ᵢ). A sensitivity analysis testing alternative weighting schemes (primary, proportional, and equal) confirmed that spatial and temporal WQI patterns were insensitive to moderate variations in weights (Figures S1a, S1b). WQI classes are as follows: excellent (80–100), good (65–79), medium (50–64), bad (25–49), and very bad (0–24).

**Table 1:** Rescaled parameter weights for the modified NSF Water Quality Index, with faecal coliform excluded and its weight redistributed proportionally across the eight retained parameters

Parameter	Original weight	Rescaled weight	Rationale for inclusion
Dissolved oxygen	0.170	0.200	Critical for fish respiration; hypoxia (< 4 mg/L) causes mortality
pH	0.120	0.140	Regulates metabolic processes; extreme values (pH < 6 or > 9) are lethal
BOD₅	0.100	0.120	Indicates organic pollution; high BOD depletes oxygen
T~w~	0.100	0.120	Tropical species are sensitive to thermal stress above 32 °C
Nitrates (NO₃-N)	0.100	0.120	Eutrophication indicator; > 5 mg/L triggers algal blooms
Phosphate (PO₄-P)	0.080	0.100	Limiting nutrient; > 0.1 mg/L promotes harmful algae
Turbidity	0.080	0.100	Affects light penetration; high turbidity reduces prey detection and promotes the growth of harmful algae
Conductivity	0.100	0.120	Proxy for dissolved solids; > 1000 μS/cm stresses freshwater fish
Total	0.85	1.00	Faecal coliform excluded (0.15 weight redistributed)

### Statistical analyses

Differences in WQI across dams, habitat types, and seasons were assessed using Kruskal–Wallis tests with Dunn’s post-hoc comparisons and Benjamini–Hochberg false-discovery-rate (FDR) correction (Benjamini & Hochberg, 1995). Multivariate water-quality patterns were characterised using Bray–Curtis dissimilarity (Bray & Curtis, 1957) on log(*x* + 1)-transformed data, permutational multivariate analysis of variance (PERMANOVA; 999 permutations; Anderson, 2017), and non-metric multidimensional scaling (NMDS) ordination (stress < 0.2).

Directional climate–water-quality relationships were evaluated using pairwise Granger causality tests (Granger, 1969), examining whether each of five climate drivers (atmospheric pressure, rainfall, relative humidity, air temperature, and windspeed) improved 1-month-ahead prediction of each of eight water-quality parameters (BOD, DO, EC, NO₃-N, pH, PO₄-P, turbidity, and water temperature) across all combinations of dam, habitat type, and season (2 × 3 × 2 strata = 12 strata; 480 tests in total). Prior to testing, each time series was assessed for stationarity using the Augmented Dickey–Fuller (ADF) test (Dickey & Fuller, 1979); non-stationary series were first-differenced. Lag length was fixed at 1 month, with admissibility verified by minimising the Akaike Information Criterion (AIC) subject to a degrees-of-freedom constraint (maximum lag = ⌊(*n* − 2)/3⌋), ensuring valid F-statistics given seasonal subgroup sizes of *n* = 5–7. Multiple testing was controlled using Benjamini–Hochberg FDR correction applied across all 480 tests. Granger causality reflects predictive temporal precedence rather than mechanistic causation. All analyses were conducted in R version 4.5.3 using the lmtest and tseries packages.

Variance partitioning via partial redundancy analysis (RDA) quantified the unique adjusted *R*^2^ contributions of spatial (dam identity, habitat type), temporal (season, month), and land-use (agricultural, urban, and forest cover) predictor sets to multivariate water-quality variation, using the vegan package (v. 2.6–4) in R 4.5.1 (Legendre & Legendre, 2012).

Six complementary changepoint detection algorithms were applied to identify abrupt transitions in water-quality time series: pruned exact linear time (PELT), binary segmentation, variance-based detection, visual inspection of abrupt transitions, cumulative sum (CUSUM) screening, and Bayesian changepoint analysis (Zeileis et al., 2003; Killick & Eckley, 2014; Castillo-Mateo, 2022). Consensus abrupt transitions were defined as detection by two or more methods within ± 1 month. Detection frequencies across parameters were used to rank candidate sentinel indicators for cost-effective monitoring.

## Results

### Water quality characterisation

Across 72 samples collected from April 2024 to March 2025, the overall mean WQI was 65.35 (median 65.20; SD 4.17), placing the system in the medium quality class (Table 2). WQI values ranged from a site-mean low of 64.64 at Kainji to a site-mean high of 66.06 at Jebba and from a monthly low of 59.95 in February to a monthly high of 71.54 in May.

**Table 2:** Descriptive statistics of the Water Quality Index (WQI) overall and by dam, habitat, and season. *N*, sample size; SD, standard deviation. Values for dam and habitat represent aggregated monthly means; seasonal values aggregate across all sites

Category	Sub-category	N	Mean WQI	Median WQI	SD WQI
Overall		72	65.35	65.20	4.17
By dam	Jebba	36	66.06	65.66	3.84
	Kainji	36	64.64	64.68	4.41
By habitat	Ecotonal	24	65.17	64.42	3.81
	Lacustrine	24	65.78	65.82	4.19
	Riverine	24	65.11	65.58	4.60
By season	Dry	48	65.60	65.66	4.41
	Wet	24	64.85	64.36	3.67

### WQI variation by dam and habitat type

**Fig. 2:**
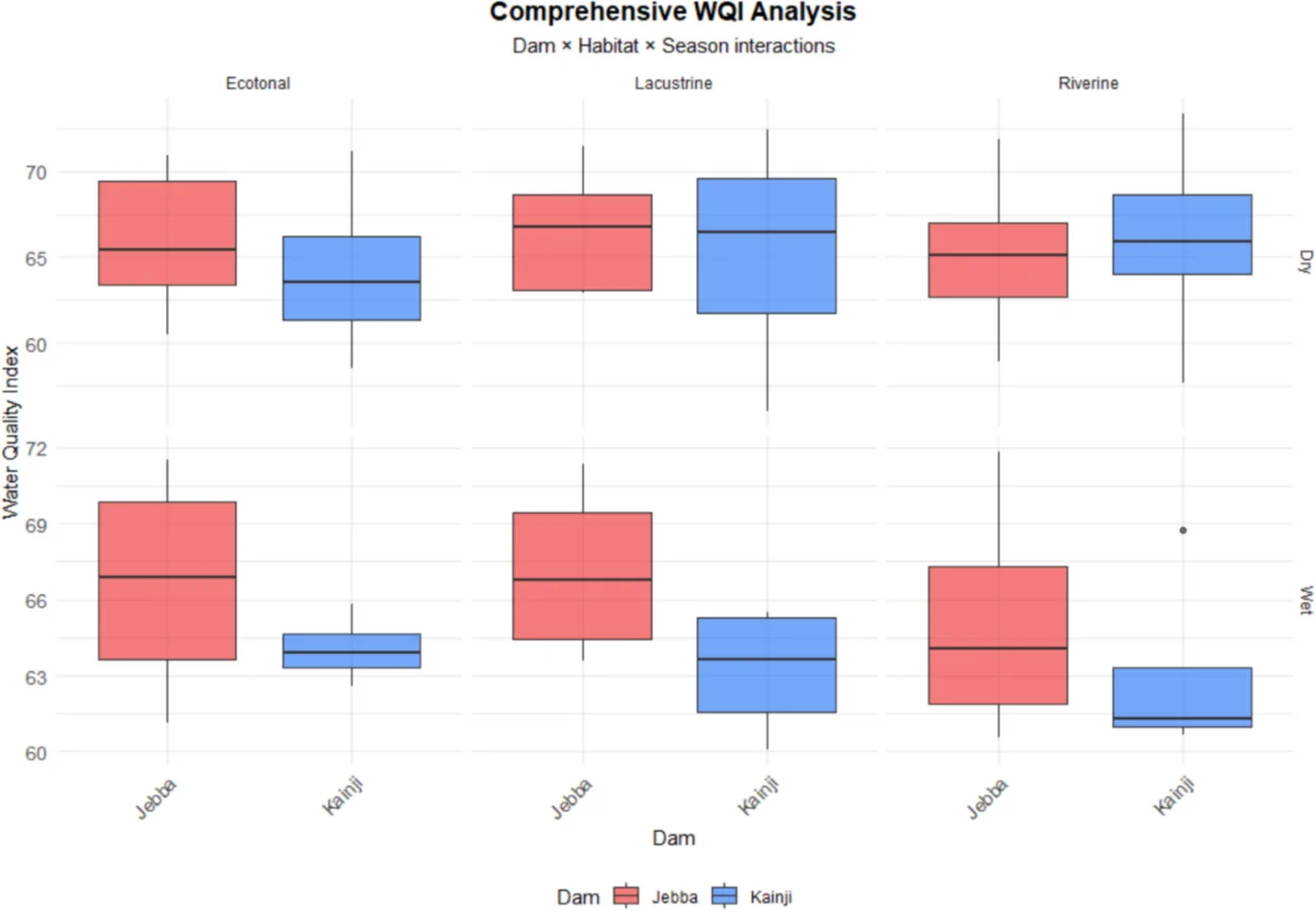
Interaction effects of dam, habitat, and season on WQI. Error bars represent the standard deviation of the mean

Mean WQI at Jebba Dam (66.06) was slightly higher than at Kainji (64.64), a difference of 1.42 units. Habitat-level differences were smaller still, with lacustrine zones recording the highest mean WQI (65.78), followed by ecotonal (65.17) and riverine (65.11) zones—a range of 0.67 units across habitat types (Fig. 2). Kruskal–Wallis tests indicated no statistically significant differences in WQI across dams (*H*(1) = 1.83, *p* = 0.176) or habitat types (*H*(2) = 0.41, *p* = 0.814) after Benjamini–Hochberg FDR correction. Together, spatial variation amounted to less than 1.5 WQI units across dams and less than 1 unit across habitats.

### Seasonal and monthly variation

**Fig. 3:**
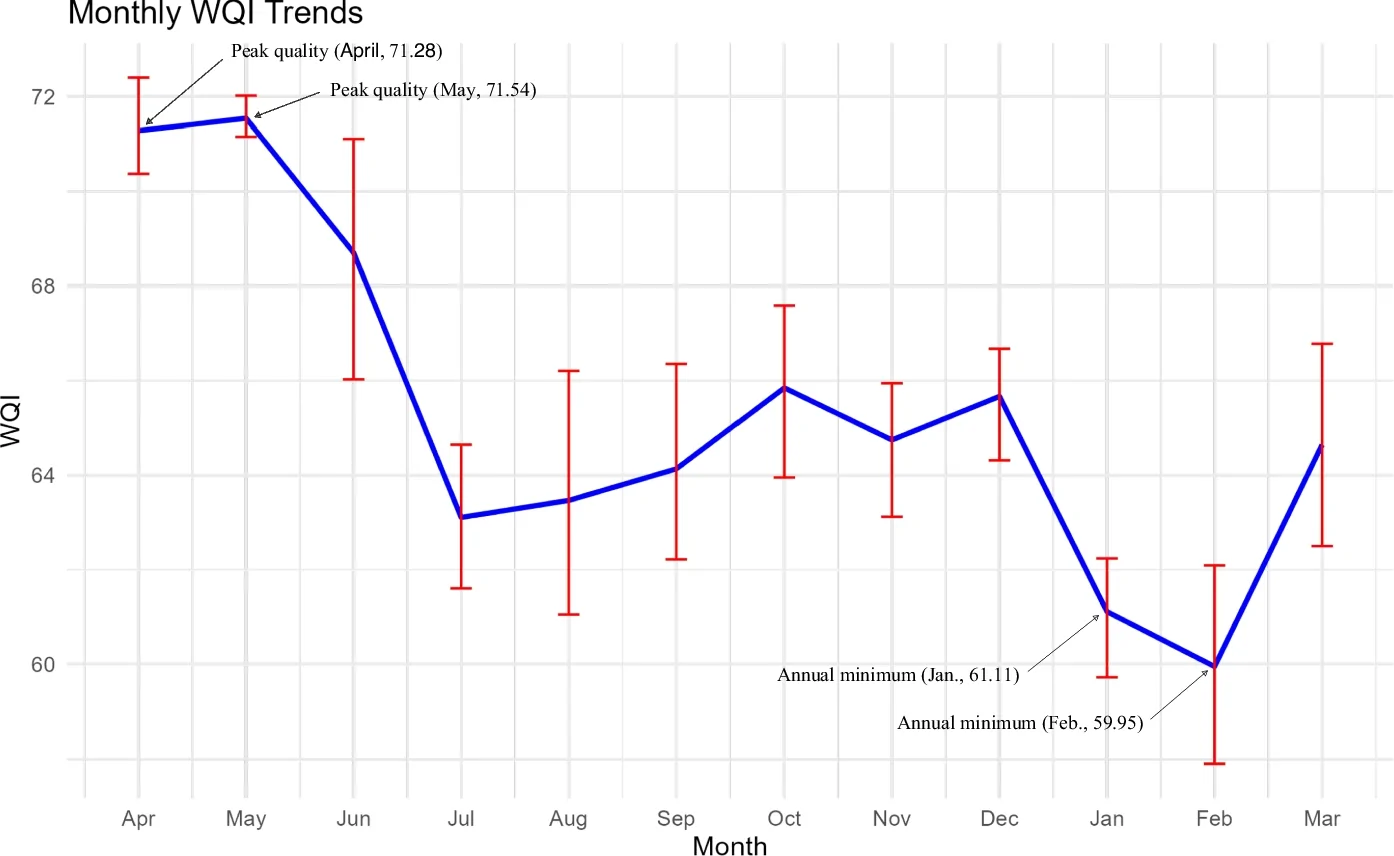
Monthly mean WQI from April 2024 to March 2025. Red error bars represent standard deviation. The peak quality (May) and the annual minimum (February) are labelled

Mean WQI was marginally higher in the dry season (65.60) than in the wet season (64.85; Fig. 2). The monthly WQI trajectory showed a more detailed pattern (Fig. 3): values peaked in April and May (71.28 and 71.54, respectively), declined sharply in June and July (68.71 and 63.11) with the onset of the wet season, remained comparatively depressed through the wet-season months, then partially recovered in the early dry season before declining again in January and February (61.11 and 59.95). February recorded the annual minimum WQI of 59.95, falling below the good/medium threshold. The total monthly range was 11.59 units, approximately eight times the spatial range observed across dams (1.42 units) and 17 times the range across habitat types (0.67 units).

### Bimodal distribution of water quality

**Fig. 4:**
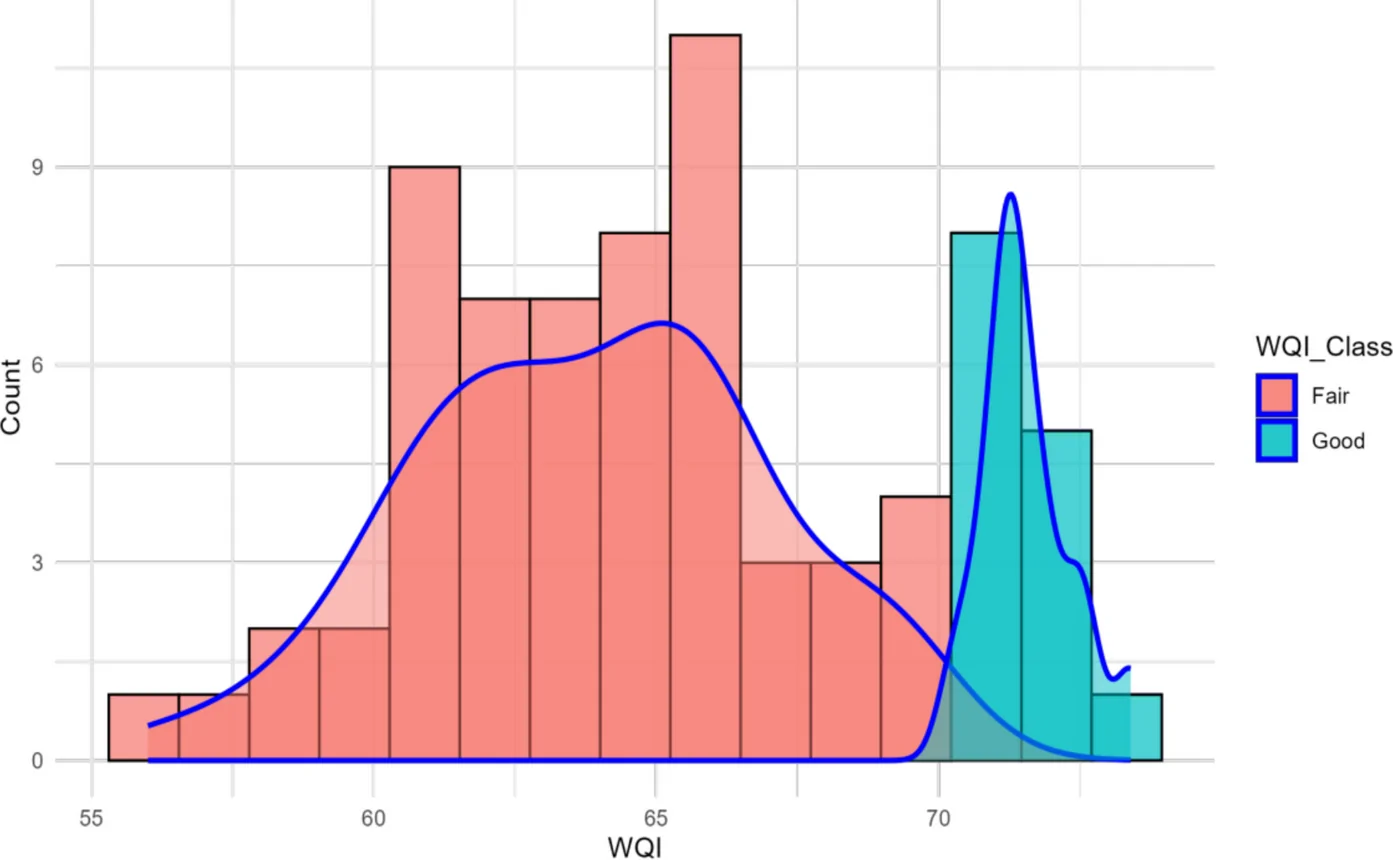
Distribution of WQI values across all 72 samples. The bimodal structure (a narrow, high-quality peak in green and a broader distribution in red) reflects two distinct seasonal water-quality regimes

The distribution of WQI values across all 72 samples showed an apparent bimodal structure (Fig. 4), with a narrow secondary peak centred around 71–72 and a broader primary distribution centred around 64–65. The narrow peak corresponds to samples collected in April and May, which recorded the lowest within-month standard deviations of the study period (April SD = 1.39; May SD = 0.61), indicating high spatial homogeneity across sites during those months. The broader distribution encompasses the remaining 10 months, which showed consistently lower and more variable WQI values.

### Granger causality analysis

**Fig. 5:**
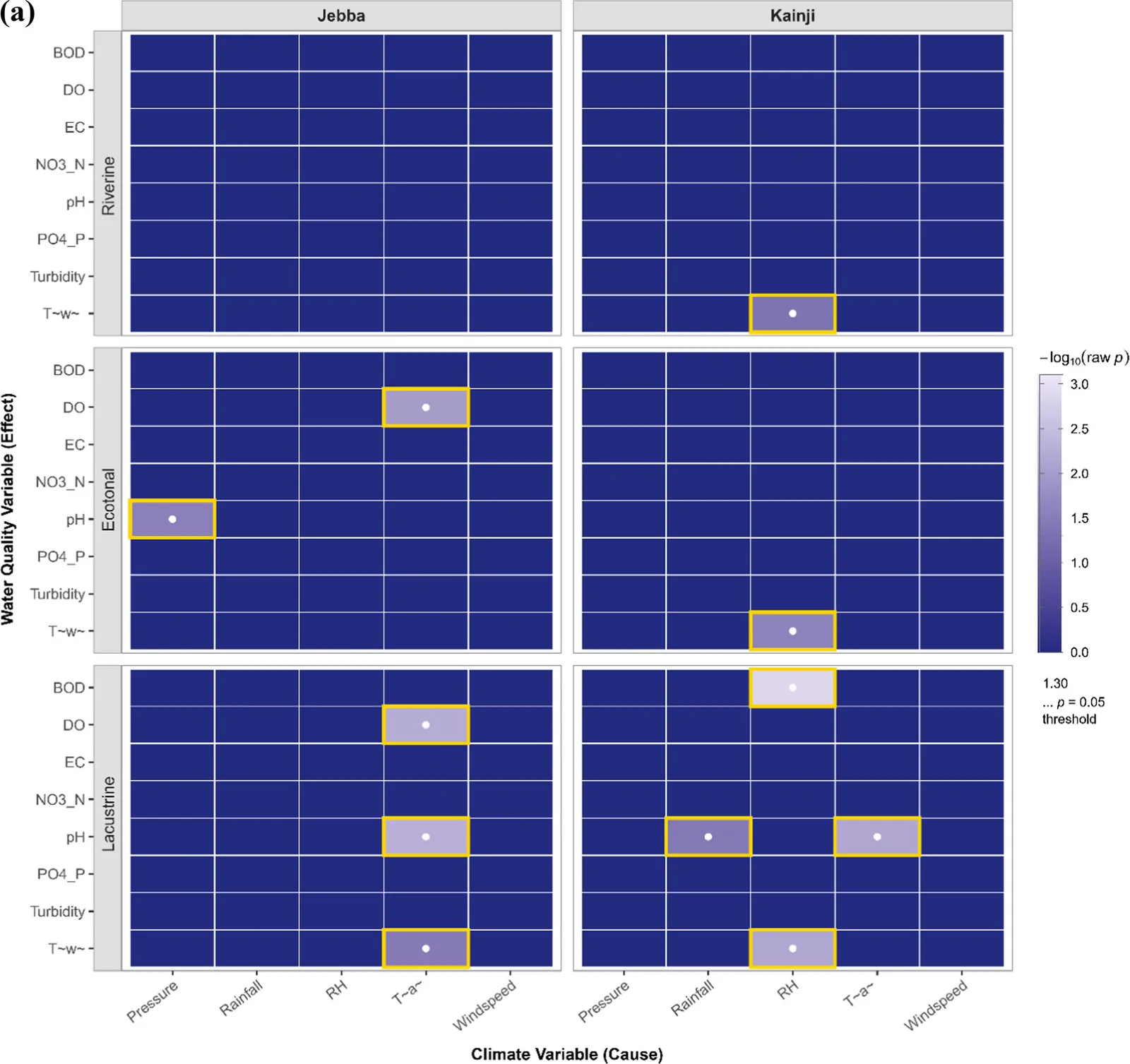

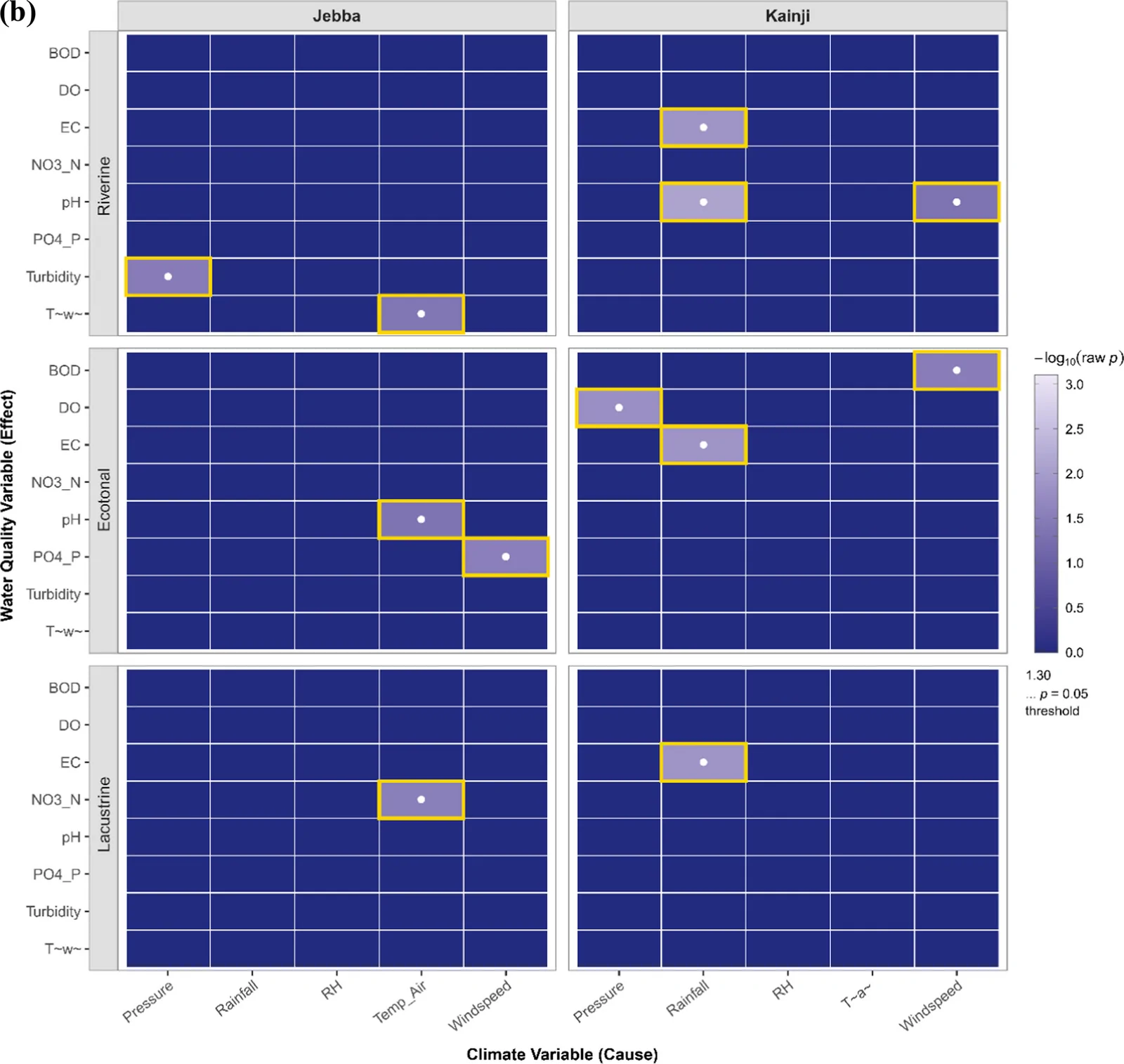
**a** Granger causality heatmap for the wet season (April–October) across the Kainji–Jebba reservoir cascade. Colour intensity reflects −log₁₀(raw *p*-value), with darker blue indicating weaker evidence of predictive association between climate drivers (*x*-axis) and water-quality parameters (*y*-axis). Yellow borders denote near-significant relationships (raw *p* < 0.05, *n* = 11). Panels are stratified by dam (Jebba, Kainji) and habitat type (Riverine, Ecotonal, Lacustrine). No relationship remained statistically significant after Benjamini–Hochberg false-discovery-rate correction (all adjusted *p* > 0.05). The dashed marker on the colour scale corresponds to the significance threshold (−log₁₀ = 1.30; *p* = 0.05). Lag = 1 month throughout; series stationarity ensured via Augmented Dickey–Fuller test with first-differencing where required. The figure forms part of the complete 480-test analysis (2 dams × 3 habitats × 2 seasons × 5 climate drivers × 8 water-quality parameters); full results are provided in Table S1. **b** Granger causality heatmap for the dry season (November–March) across the Kainji–Jebba reservoir cascade. Colour intensity reflects −log₁₀(raw *p*-value), with darker blue indicating weaker evidence of predictive association between climate drivers (*x*-axis) and water-quality parameters (*y*-axis). Yellow borders denote near-significant relationships (raw *p* < 0.05, *n* = 12). Panels are stratified by dam (Jebba, Kainji) and habitat type (Riverine, Ecotonal, Lacustrine). No relationship remained statistically significant after Benjamini–Hochberg false-discovery-rate correction (all adjusted *p* > 0.05). The dashed marker on the colour scale corresponds to the significance threshold (−log₁₀ = 1.30; *p* = 0.05). Lag = 1 month throughout; series stationarity ensured via Augmented Dickey–Fuller test with first-differencing where required. The forms part of the complete 480-test analysis (2 dams × 3 habitats × 2 seasons × 5 climate drivers × 8 water-quality parameters); full results are provided in Table S1

No statistically significant relationships between climate variables and water-quality parameters were detected after Benjamini–Hochberg FDR correction across all 480 stratified tests (5 climate drivers × 8 water-quality parameters × 12 strata; Fig. 5). Twenty-three near-significant relationships were identified at unadjusted *p* < 0.05 (Table 3), with adjusted *p*-values ranging from 0.643 to 0.955, confirming that none survived multiple-comparison correction. The strongest raw-*p* signal was observed for relative humidity predicting BOD at Kainji Lacustrine during the wet season (unadjusted *p* = 0.0014; adjusted *p* = 0.643). Air temperature accounted for several near-significant relationships, including predictions of pH, dissolved oxygen, nitrate, and water temperature across multiple strata, particularly in lacustrine and ecotonal habitats during the wet season. Rainfall showed repeated near-significant associations with electrical conductivity across all three Kainji habitats during the dry season (unadjusted *p* = 0.0135 in each case; adjusted *p* = 0.647). These relationships are therefore reported as exploratory findings from short stratified monthly time series (*n* = 5–7 per stratum) and should be interpreted as hypotheses for future confirmatory analysis rather than as evidence of directional causation.

**Table 3:** Near-significant Granger causality relationships from climate drivers to water-quality parameters across Jebba and Kainji, showing unadjusted and FDR-adjusted *p*-values

Site	Habitat	Season	Cause	Effect	Lag	Unadjusted *p*	Adj_P	Notes
Kainji	Lacustrine	Wet	RH	BOD	1	0.001	0.643	Levels
Jebba	Lacustrine	Wet	T~a~	pH	1	0.005	0.643	First-differenced
Jebba	Lacustrine	Wet	T~a~	DO	1	0.006	0.643	First-differenced
Kainji	Lacustrine	Wet	RH	T~w~	1	0.007	0.643	Levels
Kainji	Lacustrine	Wet	T~a~	pH	1	0.007	0.643	First-differenced
Kainji	Riverine	Dry	Rainfall	pH	1	0.008	0.643	Levels
Jebba	Ecotonal	Wet	T~a~	DO	1	0.010	0.647	First-differenced
Kainji	Riverine	Dry	Rainfall	EC	1	0.013	0.647	Levels
Kainji	Ecotonal	Dry	Rainfall	EC	1	0.013	0.647	Levels
Kainji	Lacustrine	Dry	Rainfall	EC	1	0.013	0.647	Levels
Kainji	Ecotonal	Dry	Pressure	DO	1	0.016	0.714	Levels
Kainji	Ecotonal	Wet	RH	T~w~	1	0.025	0.907	Levels
Jebba	Ecotonal	Wet	Pressure	pH	1	0.028	0.907	First-differenced
Jebba	Ecotonal	Dry	Windspeed	PO4_P	1	0.030	0.907	Levels
Jebba	Lacustrine	Dry	T~a~	NO3_N	1	0.030	0.907	Levels
Kainji	Ecotonal	Dry	Windspeed	BOD	1	0.032	0.907	Levels
Jebba	Riverine	Dry	Pressure	Turbidity	1	0.033	0.907	Levels
Jebba	Lacustrine	Wet	T~a~	T~w~	1	0.034	0.907	First-differenced
Kainji	Lacustrine	Wet	Rainfall	pH	1	0.036	0.907	First-differenced
Jebba	Riverine	Dry	T~a~	T~w~	1	0.040	0.955	Levels
Jebba	Ecotonal	Dry	T~a~	pH	1	0.045	0.955	Levels
Kainji	Riverine	Wet	RH	T~w~	1	0.045	0.955	Levels
Kainji	Riverine	Dry	Windspeed	pH	1	0.047	0.955	Levels

The 23 Granger causality relationships with raw *p* < 0.05 identified in the exploratory screening analysis across 480 stratified tests (2 reservoirs × 3 habitats × 2 seasons × 5 climate drivers × 8 water-quality parameters). None of these relationships remained statistically significant after Benjamini–Hochberg false-discovery-rate correction (all adjusted *p* > 0.05; adjusted *p* range: 0.643–0.955), and they are therefore retained as hypotheses for future testing with longer time series rather than as evidence of directional causation. Lag = 1 month throughout. “Notes” indicates whether the analysis was conducted on the original series (“Levels”) or on the first-differenced series following Augmented Dickey–Fuller stationarity testing. Full Granger test results are provided in Table S1

### Variance partitioning

**Fig. 6:**
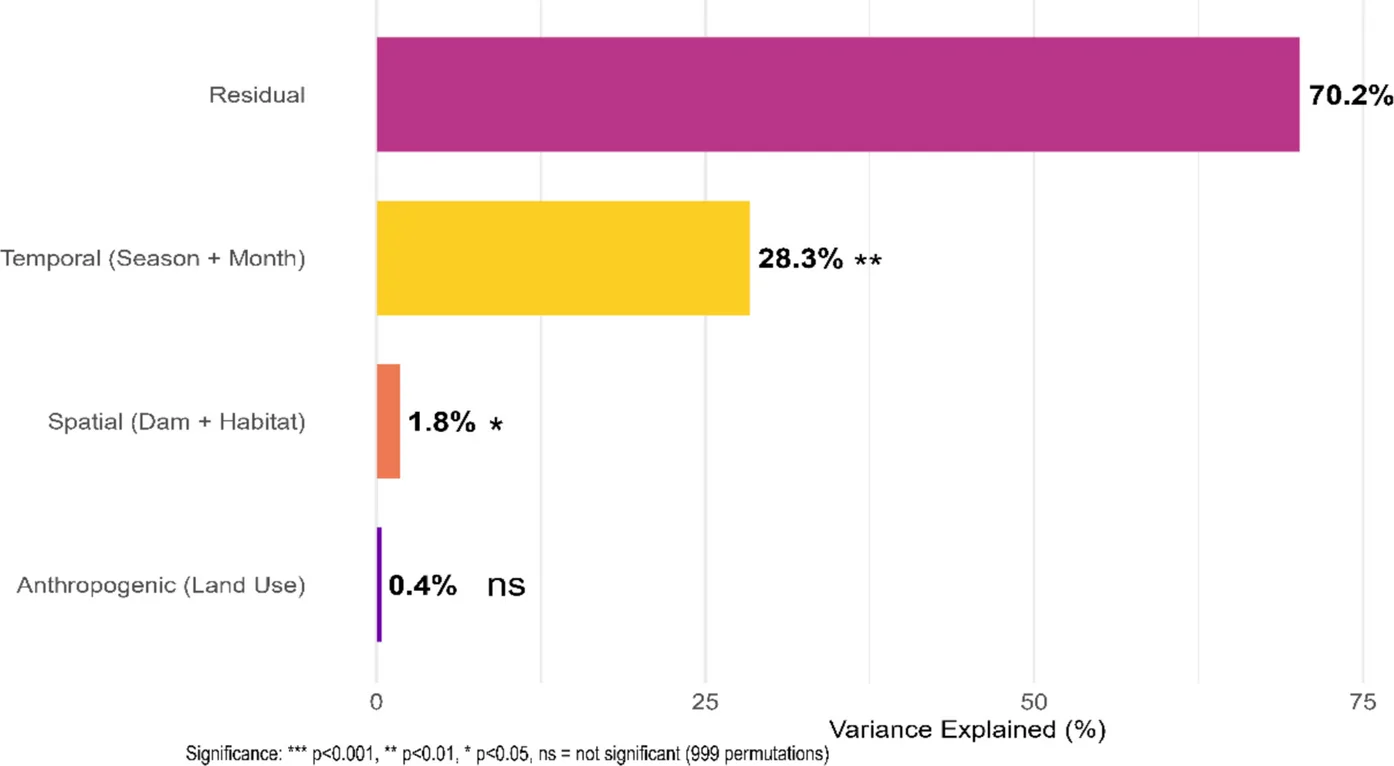
Variance partitioning of water quality (adjusted *R*^2^ from conditional RDA, *n* = 72). Temporal factors dominate. ***p < 0.001; *p < 0.05; ns, not significant

**Fig. 7:**
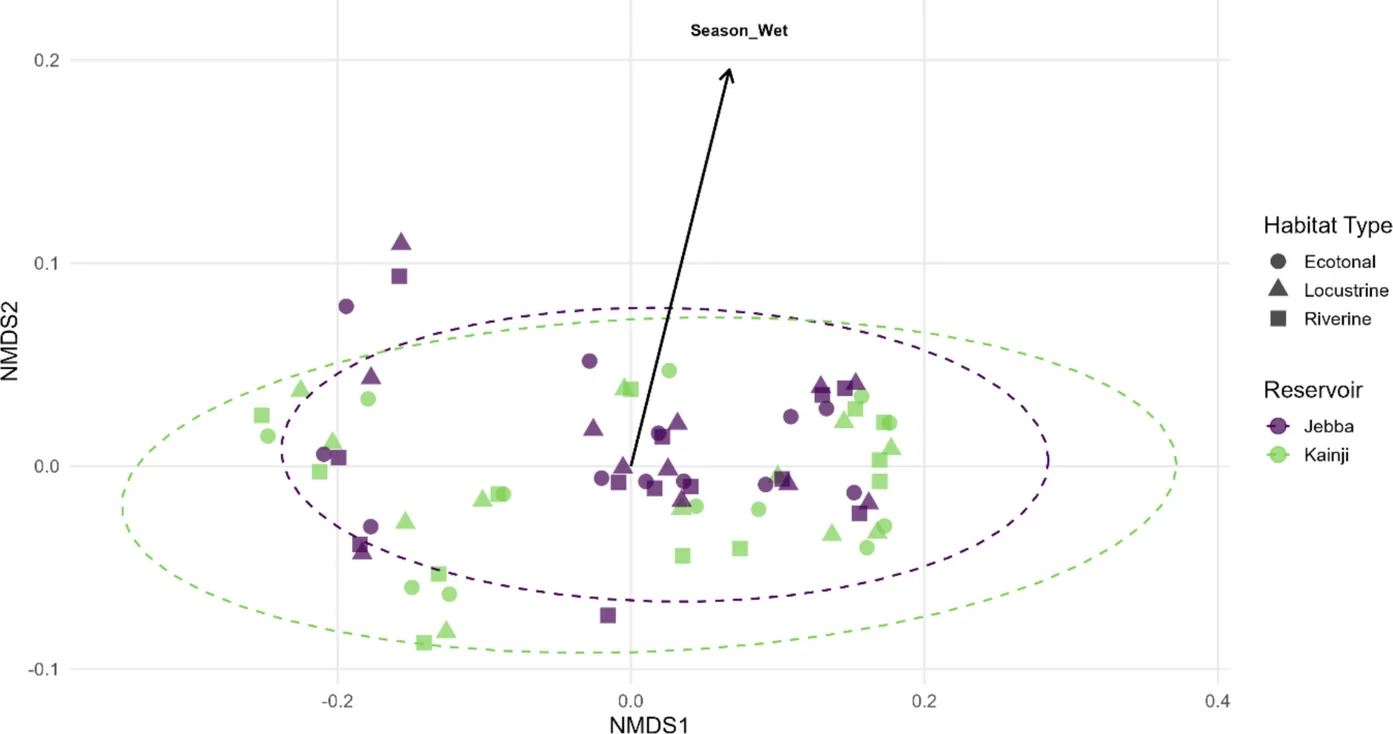
NMDS ordination of water quality parameters by season and reservoir (stress = 0.059). Points represent individual samples, coloured by season and shaped by reservoir. Ellipses represent 95% confidence intervals

Temporal factors (season and month) explained 28.3% of the variance in multivariate water-quality composition (adjusted *R*^2^; *F* = 14.32, *p* = 0.001; Fig. 6, Table S2). Spatial factors (dam identity and habitat type) explained 1.8% of the variance and remained statistically significant (*F* = 1.85, *p* = 0.038), whereas land-use predictors showed no significant independent contribution (0.4%; *p* = 0.303). Within the constraints of the present sampling design, temporal variation across six sites in two reservoirs explained a substantially larger proportion of water-quality variance than spatial factors. This result should be interpreted in light of the limited spatial extent of sampling, which may underestimate spatial gradients detectable under broader coverage. NMDS ordination (stress = 0.059) visually confirmed strong seasonal structuring, with wet-season samples distinctly separated from dry-season samples along axis 2, while dam identity and habitat type were secondary organising factors (Fig. 7).

### Changepoint detection and sentinel parameters

**Fig. 8:**
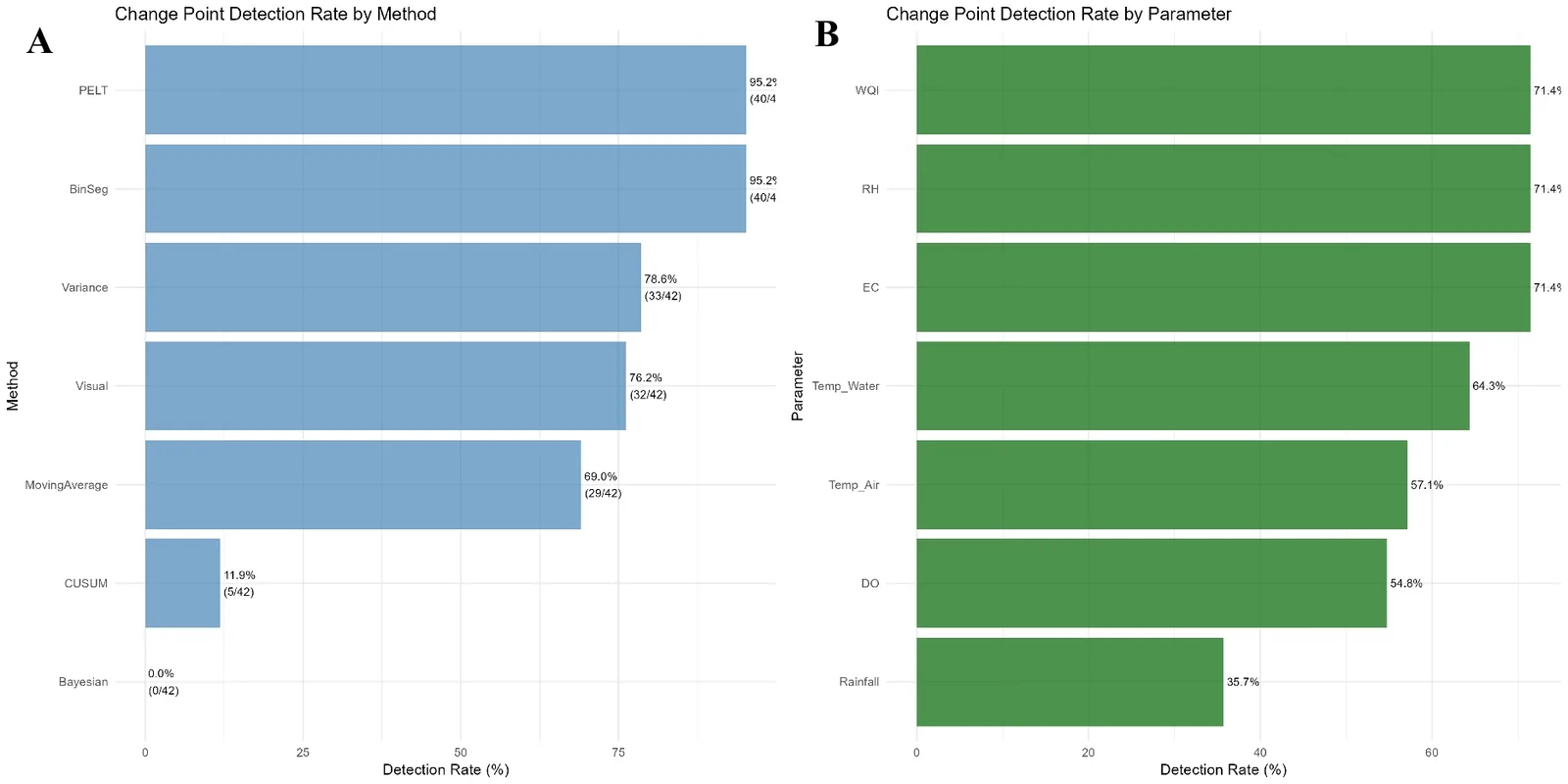
Change-point detection rates by **A** statistical method and **B** environmental parameter. PELT and BinSeg achieved the highest detection rates (95%); WQI, EC, and RH were most frequently identified with abrupt transitions (71.4% each). Numbers in parentheses indicate total analyses per category (*n* = 42)

**Fig. 9:**
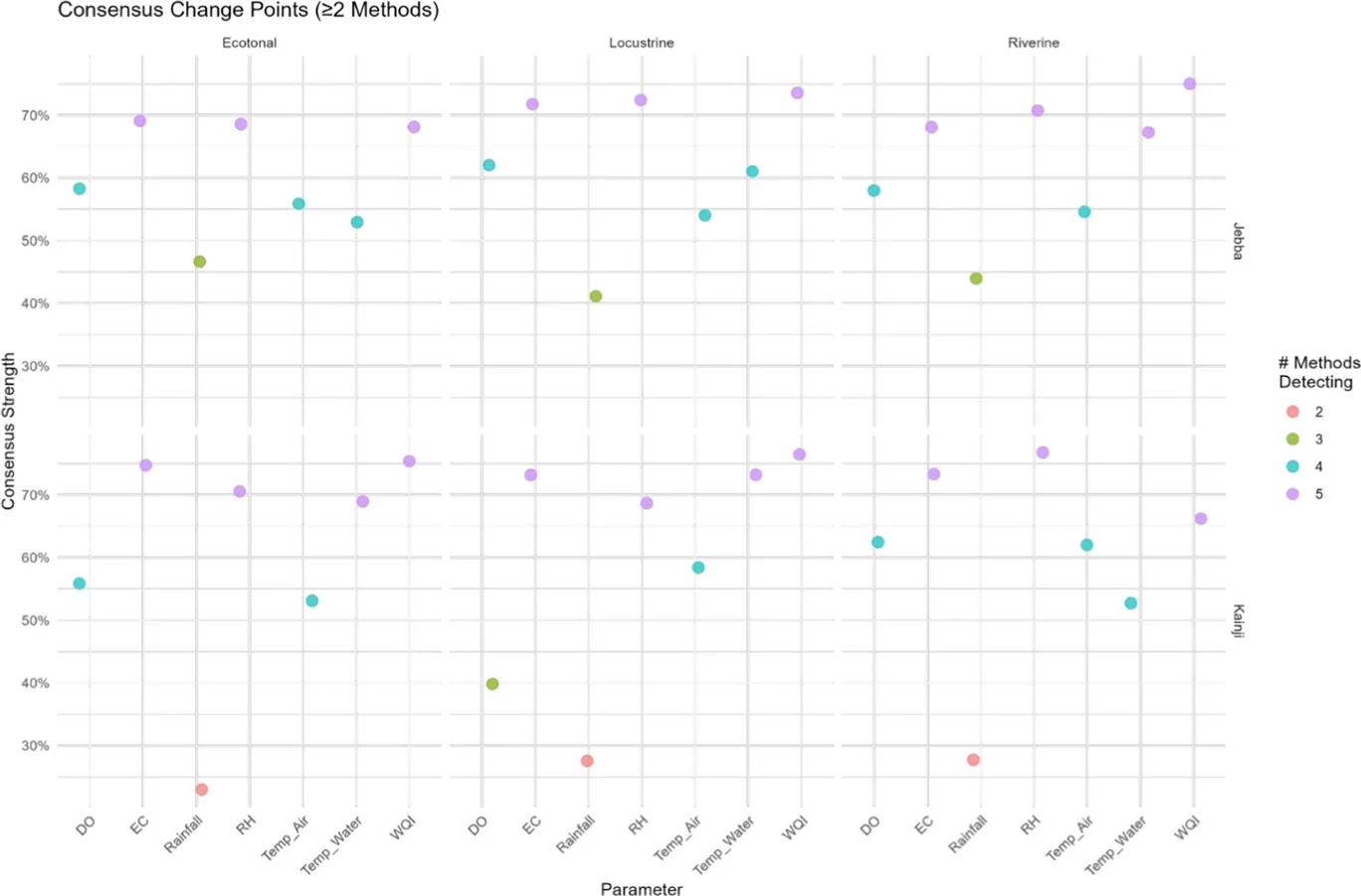
Consensus changepoint analysis showing locations and confidence levels of statistically significant abrupt transitions across habitats. Colour intensity indicates consensus strength (2–5 methods agreeing)

**Fig. 10:**
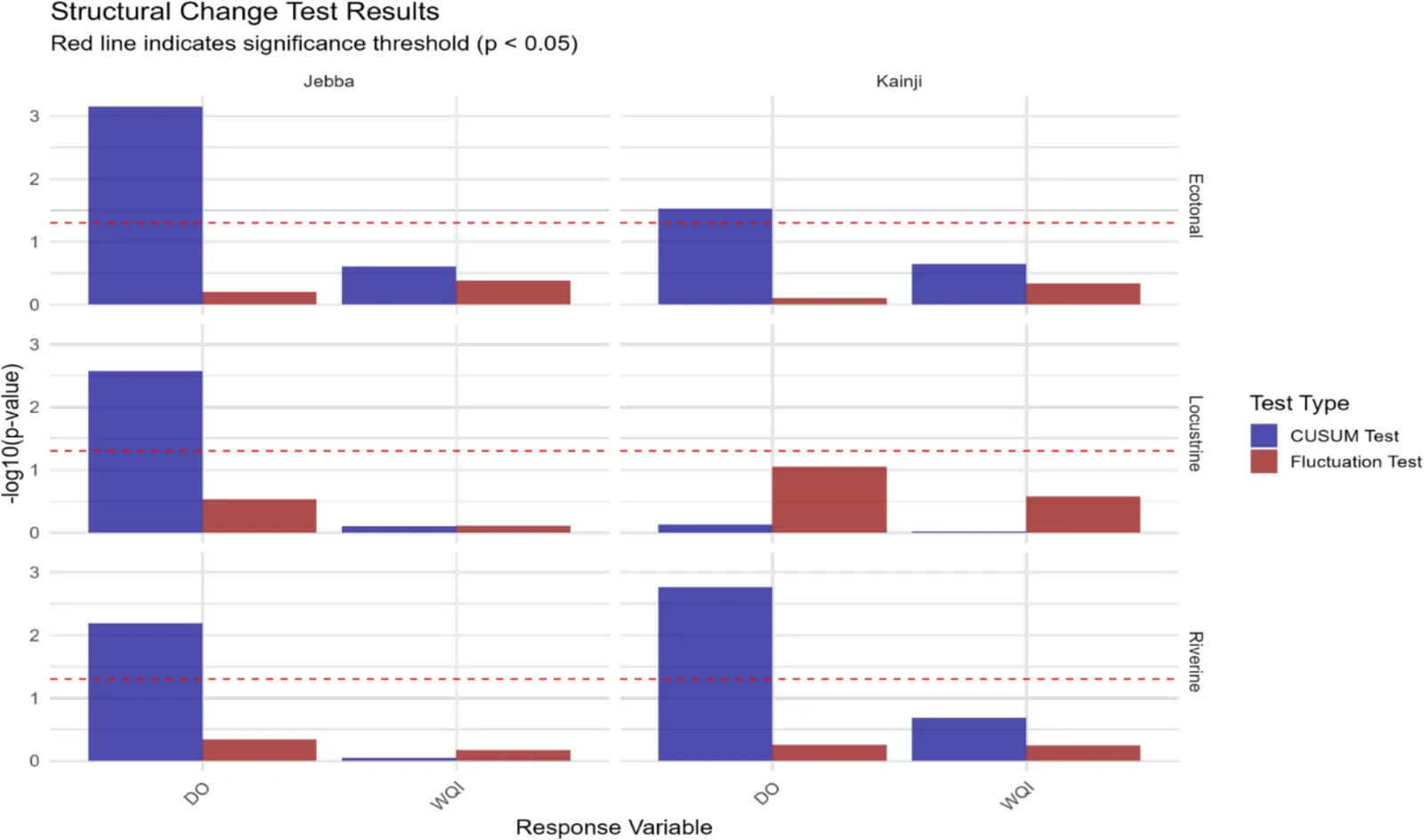
Structural change test results (CUSUM and fluctuation tests) for dissolved oxygen and WQI at Jebba and Kainji across three habitats. Bars above the dashed line indicate significance at *p* < 0.05 (−log10 scale)

PELT and binary segmentation achieved the highest detection rates across the six algorithms (95.2% each), while CUSUM achieved 11.9%, and the Bayesian approach detected no abrupt transitions (0.0%; Fig. 8). WQI, electrical conductivity, and relative humidity showed the highest consensus detection rates (71.4% each; Table 4), with consensus defined as detection by two or more methods within ± 1 month. Water temperature (64.3%), air temperature (57.1%), and dissolved oxygen (54.8%) showed moderate detection rates, whereas rainfall recorded the lowest rate (35.7%). These three parameters: WQI, EC, and RH, are identified as candidate sentinel parameters for water-quality monitoring in the cascade, although the role of RH warrants further investigation before operational adoption. Consensus changepoint analysis indicated that abrupt transitions were earlier and more frequent in riverine environments than in lacustrine systems (Fig. 9). Independent structural-change validation using CUSUM and fluctuation tests corroborated abrupt transitions in dissolved oxygen and WQI across habitats, with particularly strong signals in ecotonal and riverine dissolved-oxygen series at Kainji (Fig. 10).

**Table 4:** Changepoint detection rates for water quality and environmental parameters across all sites and methods. Consensus is defined as detection by ≥ 2 methods within ± 1 month

Parameter	Total analyses	Detections	Detection rate (%)
Water Quality Index (WQI)	42	30	71.4
Electrical conductivity (EC)	42	30	71.4
Relative humidity (RH)	42	30	71.4
Water temperature (T~w~)	42	27	64.3
Air temperature (T~a~)	42	24	57.1
Dissolved oxygen (DO)	42	23	54.8
Rainfall	42	15	35.7

## Discussion

Within the constraints of the present sampling design, temporal variation explained a substantially larger proportion of water-quality variance than spatial factors across the Kainji–Jebba reservoir cascade. Seasonal and monthly processes accounted for 28.3% of multivariate water-quality variance, compared with 1.8% for spatial factors and 0.4% for land-use predictors. These results are broadly consistent with the flood pulse concept, in which seasonal hydrological cycles may drive ecosystem dynamics more strongly than the longitudinal gradients central to temperate models such as the River Continuum Concept (Vannote et al., 1980; Junk et al., 1989; Tockner & Stanford, 2002). This interpretation should, however, be considered in light of the limited spatial extent of sampling: six sites across two reservoirs may underestimate spatial gradients that would be detectable with broader coverage, and the comparatively weak spatial signal may partly reflect the resolution of the sampling design rather than a genuine absence of spatial structuring in the system.

### Spatial patterns: weak zonation in a connected system

The small but statistically significant spatial differences observed—slightly higher WQI at Jebba (66.06) than at Kainji (64.64), and marginally better quality in lacustrine than in riverine habitats—are interpretable in terms of local hydrological position. Kainji, situated approximately 100 km upstream, is the first reservoir to receive the sediment-laden white flood from upstream agricultural catchments, which may carry elevated concentrations of suspended material, nutrients, and organic pollutants mobilised from intensively farmed soils during the initial wet-season flush (Adeogun et al., 2018). Jebba may benefit from partial gravitational settling and dilution as water passes through the upper reservoir before reaching the lower dam, consistent with the documented self-purification capacity of reservoir systems (Irenosen et al., 2012; Adegbehin et al., 2016). Lacustrine zones showed marginally higher WQI than riverine and ecotonal zones, a pattern potentially consistent with longer water residence times, which allow suspended material to settle and soluble pollutants to disperse, as reported in comparable tropical reservoir studies (Wetzel, 2001; Okafor et al., 2025). These spatial differences are nevertheless minor in magnitude, amounting to less than 1.5 WQI units across dams and less than 1 unit across habitat types, and are operationally less important than the seasonal signal for monitoring design. Comparable Afrotropical and tropical reservoir studies have reported similarly modest spatial gradients relative to temporal variation: Faye et al. (2023) found that seasonal hydrology dominated water-quality structuring at Manantali Dam in West Africa, while Medupe and Letshwenyo (2024) reported analogous seasonal dominance in a southern African regulated impoundment.

### Seasonal and monthly variation: runoff pulses outweigh dilution

The overall mean WQI of 65.35 places the Kainji–Jebba cascade in the Medium quality class under the NSF framework (Brown et al., 1970), a classification consistent with the CCME fair-quality threshold (Canadian Council of Ministers of the Environment, 2001) and Balamurugan et al.’s (2020) moderate category. The February mean WQI of 59.95 falls below the NSF good/medium boundary and approaches the lower end of the medium class, a level associated in the broader literature with conditions that may stress sensitive fish species and impair ecosystem function during sustained exposure (Chidiac et al., 2023). Comparable seasonal minima have been reported in other regulated tropical reservoirs; for example, Wiranegara et al. (2023) documented similarly depressed dry-to-wet transition WQI values at Cirata Reservoir, Indonesia, attributing them to first-flush runoff dynamics analogous to those identified here.

The counterintuitive finding that dry-season mean WQI (65.60) exceeded wet-season mean WQI (64.85) is consistent with the dominance of runoff-driven pollution over dilution effects. During the wet season, increased rainfall may mobilise sediments, nutrients, and organic matter from agricultural and urban catchment surfaces into the reservoirs, with runoff-borne pollutant loading potentially outweighing any dilution benefit from higher discharge volumes (Medupe & Letshwenyo, 2024). The sharp WQI decline from 71.54 in May to 63.11 in July is consistent with a first-flush mechanism, whereby accumulated dry-season pollutant deposits on land surfaces are rapidly transported into the water column at the onset of rainfall before the system can assimilate the load (Wiranegara et al., 2023). The secondary deterioration in January–February, when the WQI reached the annual minimum of 59.95, coincided with the arrival of the black flood originating in the Fouta Djallon highlands and flowing through Guinea, Mali, and Niger. Although black-flood waters traverse extensive upstream floodplains where some coarse sediment is deposited, prolonged flow may transport an accumulation of dissolved solutes and fine organic material from distant catchments, producing a secondary deterioration in water quality distinct from the local wet-season signal (Nwobi-Okoye & Igboanugo, 2013). These two periods—June–July and January–February—therefore, represent the highest-risk windows for water-quality deterioration in the cascade.

### Bimodal water-quality distribution: two seasonal states

The apparent bimodal distribution of WQI values (Fig. 4) reflects the operation of two distinct seasonal water-quality states rather than a continuum of conditions. The narrow high-quality peak centred around 71–72, corresponding to April and May samples, is characterised by low within-month variability (SD = 0.61–1.39), indicating a period of high spatial homogeneity when terrestrial pollutant loads are minimal, and the reservoir system operates under relatively stable dry-season conditions. The broader, lower distribution encompasses the remaining 10 months, during which runoff-driven inputs, black-flood dynamics, and internal biogeochemical cycling produce more variable, consistently lower WQI values. The abruptness of the transition between these two states, reflected in the sharp June–July WQI decline, suggests that the onset of the wet season acts as a threshold trigger rather than a gradual forcing, a pattern consistent with pulse-driven tropical aquatic systems (Junk et al., 1989).

### Land-use effects: expressed temporally rather than spatially

The minimal unique variance attributable to land use (0.4%, *p* = 0.303) does not necessarily indicate that agricultural and urban land use are ecologically unimportant in this system. Buffer-based land-cover metrics, while computationally tractable and reproducible, do not fully capture hydrological connectivity, preferential flow pathways, or pollutant transport processes (Carvalho et al., 2019), and the limited number of sampling sites may further reduce the detectable spatial land-use signal. The synchronous WQI declines observed across all sites at the onset of the wet season, regardless of the specific land-cover composition of their individual catchment buffers, suggest that land-use effects in this system are expressed as temporally coherent, basin-wide runoff pulses rather than as persistent spatial gradients in which some sites are consistently more degraded than others (Saturday et al., 2025; Shakeri Bostanabad et al., 2025). When all sites deteriorate simultaneously, variance attributable to land use accumulates within the temporal rather than the spatial component of the partition, meaning the land-use signal is likely embedded within the 28.3% temporal fraction rather than absent from the system (Carvalho et al., 2019). The weak unique land-use fraction should therefore be interpreted as reflecting the resolution of the metric and the scale of sampling, rather than as evidence that catchment land use is ecologically inconsequential in the Upper Niger River Basin.

### Absence of climate signals: power constraints and possible nonlinearity

The absence of any statistically significant climate–water-quality relationship after Benjamini–Hochberg FDR correction across all 480 stratified Granger tests should be interpreted as a dataset-bounded, exploratory observation rather than as a definitive ecological conclusion. Linear Granger tests applied to 12-observation monthly time series have limited statistical power, and the conservative multiple-comparison correction applied across 480 tests further reduces the likelihood of detecting relationships of moderate effect size even where they exist (Zolghadr-Asli et al., 2021). This null result does not preclude the presence of climate forcing in the system; rather, it suggests that, if present, it was not detectable within the analytical constraints of the present study. Longer time series, multi-year meteorological records, and nonlinear causal methods are required before firm conclusions can be drawn about the role of climate in driving water quality in the Upper Niger River Basin.

Several mechanistic pathways may nonetheless contribute to the absence of detectable linear signals. Internal biogeochemical feedbacks, including microbial respiration, nutrient cycling, and phytoplankton dynamics, may amplify, dampen, or redirect initial meteorological forcing, thereby obscuring the original climate signal at monthly resolution (Farrell et al., 2020; Kondowe et al., 2022). Dam operations and regulated releases may impose an anthropogenically controlled hydrological regime that partially decouples water quality from meteorological drivers, particularly during managed low-flow periods (Vivan et al., 2014; Capon et al., 2021). In addition, climate–water-quality relationships in tropical reservoirs may be nonlinear or threshold-governed in ways that linear Granger tests are not designed to capture (Ye et al., 2022).

Twenty-three near-significant relationships were identified at unadjusted *p* < 0.05 (Table 3), distributed across all three habitats and both seasons, none of which survived FDR correction. The strongest signals were concentrated in lacustrine habitats during the wet season, where relative humidity showed a near-significant predictive relationship with biochemical oxygen demand at Kainji Lacustrine (*p* = 0.001), and air temperature showed near-significant associations with both pH and dissolved oxygen at Jebba Lacustrine (*p* = 0.005 and *p* = 0.006, respectively). These patterns are potentially consistent with thermally driven stratification dynamics in deep reservoir cores during wet-season warming, whereby increased air temperature may intensify thermal stratification, suppress vertical mixing, and promote hypolimnetic oxygen depletion and pH shifts through altered microbial decomposition rates (Wetzel, 2001; Farrell et al., 2020). In riverine habitats during the dry season, rainfall showed near-significant predictive associations with both pH and EC at Kainji Riverine (*p* = 0.008 and *p* = 0.013, respectively), patterns potentially consistent with intermittent rainfall events mobilising alkalinity-altering solutes and dissolved ions from catchment soils into the river channel during otherwise low-flow conditions (Rolls et al., 2012; Duong et al., 2023). The broader spatial and seasonal distribution of near-significant signals across habitats may reflect the multi-scale hydrological connectivity of the cascade, in which meteorological forcing propagates through lacustrine, ecotonal, and riverine zones via different mechanistic pathways and at different time lags. All 23 relationships are treated as hypothesis-generating observations for future testing with longer datasets and nonlinear methods such as convergent cross-mapping.

### Sentinel parameters and a tiered monitoring framework

The high consensus detection rates for WQI, EC, and RH (71.4% each) provide an evidence-based foundation for a tiered monitoring framework, although each parameter warrants specific qualification. WQI’s composite nature makes it inherently sensitive to multi-parameter deterioration, translating complex physicochemical data into a single actionable score; because it integrates dissolved oxygen, pH, turbidity, nutrients, and conductivity, abrupt transitions in any contributing parameter can propagate into detectable WQI abrupt transitions (Uddin et al., 2021; Chidiac et al., 2023). EC responds rapidly to influxes of dissolved solids from pollution events and shifts in hydrological regime, making it a reliable integrative signal of catchment-scale processes (US EPA, 2025a). The identification of RH as a candidate sentinel parameter is unexpected and should be interpreted cautiously: RH likely serves as a proxy for broader seasonal atmospheric conditions rather than a direct mechanistic driver of water-quality change, and its utility as an operational monitoring parameter warrants further investigation before adoption in management frameworks. Water temperature and dissolved oxygen showed moderate detection rates (64.3% and 54.8%, respectively) and remain useful as ecological context indicators, particularly for detecting thermal stress and hypoxia in sensitive habitat zones. Rainfall showed the lowest detection rate (35.7%), consistent with its event-driven variability and poorer fit with changepoint methods designed to detect sustained shifts in the mean (Reeves et al., 2007; Lund et al., 2023).

The multi-method consensus framework adds a practical confidence dimension for management decision-making. High agreement across four or more algorithms indicates a high-confidence abrupt transition warranting immediate investigation, whereas agreement across two or three methods provides a preliminary alert justifying intensified monitoring before a full management response is triggered. This tiered structure transforms changepoint detection from a research tool into a risk-based management instrument applicable to data-poor tropical monitoring programmes (Lund et al., 2023).

### Management implications

Three management priorities emerge from these findings. First, monitoring programmes should intensify sampling during the two highest-risk windows identified here: June–July at the onset of the wet season, when WQI declined from 71.54 to 63.11 within 2 months, and January–February during the black flood, when the annual minimum of 59.95 was recorded. Bi-weekly sampling during these windows, combined with monthly surveillance otherwise, would concentrate effort at the periods of greatest ecological risk while remaining feasible within resource-constrained programmes (Carvalho et al., 2019; Shaaban & Stevens, 2025). Second, WQI, EC, and RH could be prioritised provisionally as sentinel parameters, with water temperature and dissolved oxygen retained as core ecological context metrics; this configuration would support earlier detection at lower cost than continuous monitoring of the full parameter suite. Third, watershed-scale interventions targeting the sources of first-flush and black-flood pollution loads, including riparian buffer restoration, agricultural erosion control, and stormwater retention infrastructure, would directly address the runoff pulses responsible for the most pronounced water-quality deterioration in the cascade. Coordination between reservoir operators and water-quality monitoring programmes during identified risk windows may offer additional scope for mitigation through managed releases that optimise dilution capacity (Chiromo et al., 2016).

## Limitations

### One-year data duration

The study covers a single complete hydrological cycle (April 2024–March 2025), which is appropriate for characterising seasonal patterns, the dominant signal in tropical systems, but insufficient to distinguish persistent structural change from interannual variability. Whether the temporal patterns and sentinel parameters identified here represent stable, recurring features of the Kainji–Jebba cascade or reflect conditions specific to the 2024–2025 hydrological year cannot be determined from the present dataset. The null Granger causality result in particular should be interpreted in light of this constraint: the absence of statistically significant linear climate–water-quality relationships across 480 tests may partly reflect reduced statistical power within a 12-month time series rather than a genuine absence of climatic influence. Multi-year monitoring with a consistent sampling protocol is therefore a priority for future work.

### Limited spatial sampling

With six sites distributed across two reservoirs, the spatial sampling intensity is modest relative to the system’s scale. Formal spatial hotspot detection using Getis–Ord Gi* statistics was not feasible, as this method requires at least ten spatial units for reliable inference; spatial patterns were therefore characterised descriptively. The low unique variance attributable to spatial factors (1.8%) and land use (0.4%) should be interpreted cautiously: buffer-based land-cover metrics do not fully capture hydrological connectivity, flow pathways, or pollutant transport processes, and the limited number of sites may reduce the apparent spatial signal. Studies sampling a broader spatial extent—including tributaries and ungauged sub-catchments—may reveal stronger spatial gradients not captured here**.**

### Monthly sampling frequency

Monthly sampling resolves seasonal but not event-scale dynamics. Episodic pollution pulses, including acute agricultural runoff events, flood-driven sediment inputs, and short-duration oxygen-depletion episodes—occurring between sampling visits—are not captured in the present dataset. The two identified seasonal risk windows are based on monthly means and therefore represent conservative estimates of peak deterioration. High-frequency or event-triggered sampling during these windows would provide a more precise characterisation of pollution dynamics and sentinel-parameter behaviour.

### Application of complex analyses to a short time series

The application of Granger causality testing and multiple changepoint detection methods to 12-observation time series entails low statistical power and an elevated risk of both false-positive and false-negative outcomes. The 480 stratified Granger tests were conducted as an exploratory screening exercise to identify candidate climate–water-quality relationships for future testing with longer datasets, rather than as a confirmatory analysis; all results should therefore be interpreted accordingly. Similarly, changepoint detection rates reflect the sensitivity of each parameter to the specific hydrological transitions of the 2024–2025 cycle and may not generalise without replication. The complementary multi-method approach—using six algorithms and defining consensus as detection by two or more methods within ± 1 month—was adopted to reduce the influence of algorithm-specific assumptions and improve the robustness of the sentinel-parameter rankings, but does not eliminate uncertainty arising from the short time series. Nonlinear causal modelling methods such as convergent cross-mapping, together with vertical profiling to capture stratification dynamics, are priorities for future work that could strengthen and extend the present findings.

## Conclusions

Within the constraints of a single hydrological cycle and six sampling sites across two reservoirs, temporal variation explained a substantially larger proportion of water-quality variance than spatial factors in the Kainji–Jebba reservoir cascade, suggesting that seasonal hydrological dynamics are a more important structuring force than dam identity or habitat type in this regulated system, thereby addressing the first research question. Monthly WQI values declined from a dry-season peak of 71.54 in May to an annual minimum of 59.95 in February. This pattern was associated with two distinct runoff-pulse periods: the white flood at wet-season onset in June–July and the black flood in January–February. No climate–water-quality relationships survived Benjamini–Hochberg FDR correction across 480 stratified Granger causality tests, a null result treated as hypothesis-generating rather than conclusive given the power constraints of a 12-month dataset, and suggesting that human-modified hydrology and catchment pollution were the proximate temporal drivers of change, thereby addressing the second research question. WQI, electrical conductivity, and relative humidity each achieved the highest consensus changepoint detection rates (71.4%), identifying them as candidate sentinel parameters for cost-effective monitoring, although the role of relative humidity warrants further investigation before operational adoption, thereby addressing the third research question. Together, these findings suggest that monitoring programmes in regulated tropical reservoir cascades may benefit from prioritising targeted sampling during the two identified seasonal risk windows—June–July and January–February—rather than expanding spatial coverage, pending validation across longer time series and broader spatial extents. Beyond the Niger Basin, the integrated analytical framework combining WQI, variance partitioning, exploratory Granger causality, and multi-method changepoint detection offers a transferable template for other data-poor tropical river systems in which urgent conservation needs and limited monitoring resources must be reconciled efficiently, and in which identifying when to monitor may yield greater returns than determining where (Yates et al., 2025).

## Supplementary information

Below is the link to the electronic supplementary material.

ESM 1 (DOCX 15.0 KB)

ESM 2 (PNG 62.1 KB)

ESM 3 (PNG 36.0 KB)

ESM 4 (CSV 35.6 KB)

ESM 5 (DOCX 14.6 KB)

## Data Availability

The datasets generated during this study will be deposited in the Dryad Digital Repository upon manuscript acceptance. The DOI will be provided during the revision process.

## Code availability

All R scripts used for data analysis, variance partitioning, Granger causality testing, and changepoint detection will be made available on GitHub upon manuscript acceptance.
