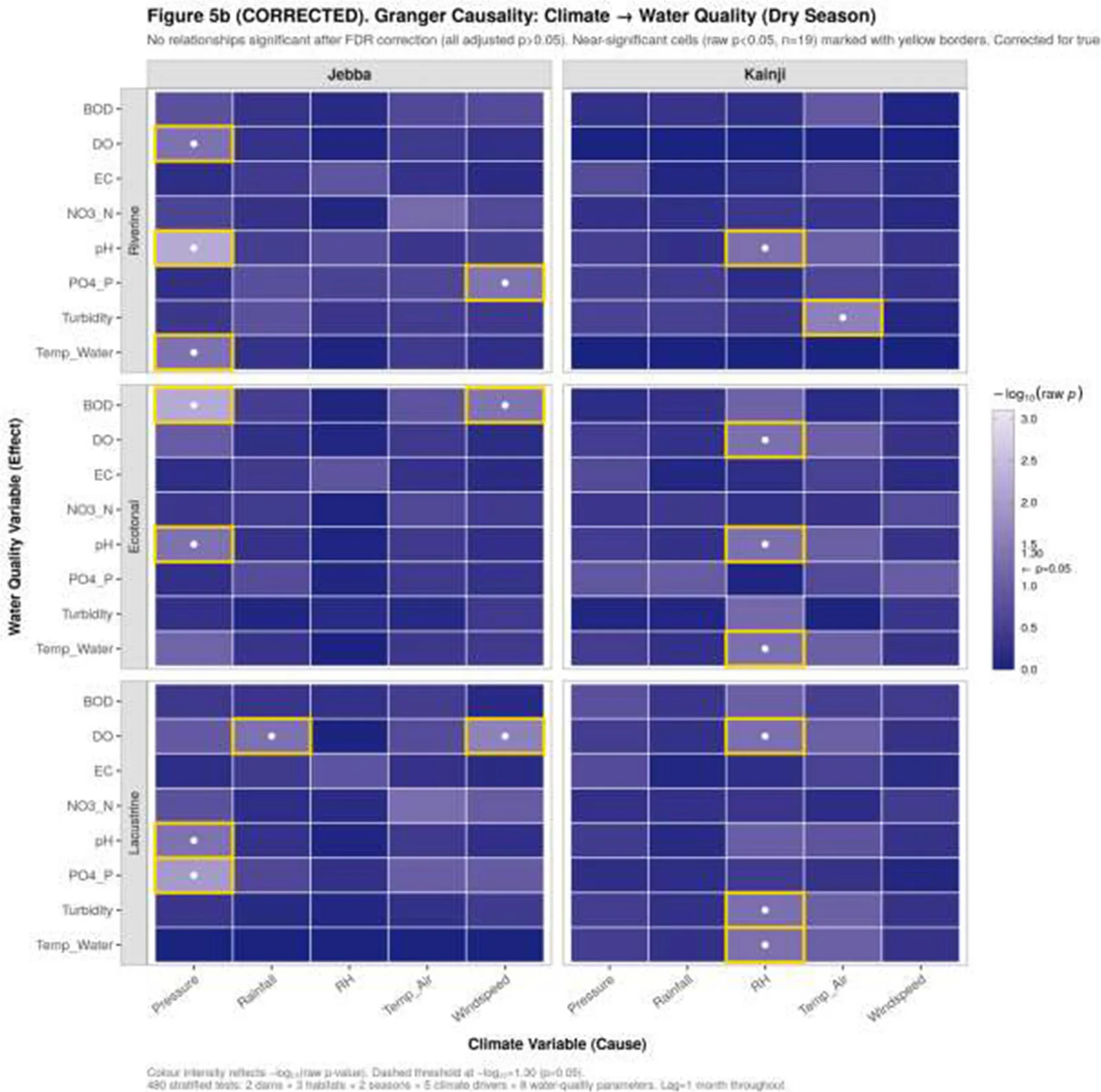# Correction to: Temporal dynamics outweigh spatial gradients in shaping water quality across a regulated Afrotropical reservoir cascade

**DOI:** 10.1007/s10661-026-16015-4

**Published:** 2026-10-21

**Authors:** Abdullahi Ibrahim, Suleiman Omeiza Eku Sadiku, Deborah Vivienne Robertson‑Andersson, Moses Okpeku

**Affiliations:** 1 Discipline of Biology, School of Agriculture and Sciences, University of KwaZulu-Natal, Westville Campus, Durban, South Africa; 2 Department of Water Resources, Aquaculture and Fisheries Technology, School of Food Science and Agricultural Technology, Federal University of Technology, Minna, Nigeria; 3 Chrysalis Training and Skills Development, Kei Mouth, South Africa

**Correction to: Environ Monit Assess (2026) 198:906**

**https://doi.org/10.1007/s10661-026-15681-8**

Unfortunately, the original version of this article contained numerical errors in two analyses found in Figure 6, Table 2, the Abstract, Results, Discussion sections, Table 3 and Figures 5a–5b.

Published and corrected values are compared below:

**Table:** 

Component	Published	Corrected	Δ pp
Temporal (Season + Month)	28.3%; F=14.32; p=0.001 (**)	33.4%; F=17.90; p=0.001 (**)	+5.1
Spatial (Dam + Habitat)	1.8%; F=1.85; p=0.038 (*)	2.0%; F=1.99; p=0.013 (*)	+0.2
Land Use (Anthropogenic)	0.4%; p=0.303 (ns)	0.5%; p=0.247 (ns)	+0.1

In the Abstract section: “28.3%” should read “33.4%”.

In the Results section, variance-partitioning paragraph: All three reported percentages and test statistics for temporal, spatial, and land-use components should be updated to the corrected values shown in the table above. The corrected Table 2 and Figure 6 are shown below.

**Table 2:** Descriptive statistics of the Water Quality Index (WQI) overall and by dam, habitat, and season. N, sample size; SD, standard deviation. Values for dam and habitat represent aggregated monthly means; seasonal values aggregate across all sites

Component	Variance_pct	F_value	p_value	Significance	
Temporal (Season + Month)	33.37754	17.9005	0.001	**	
Spatial (Dam + Habitat)	1.961409	1.993147	0.013	*	
Anthropogenic (Land Use)	0.515047	1.176495	0.247	ns	
Residual	65.17313	NA	NA		

**Figure:**
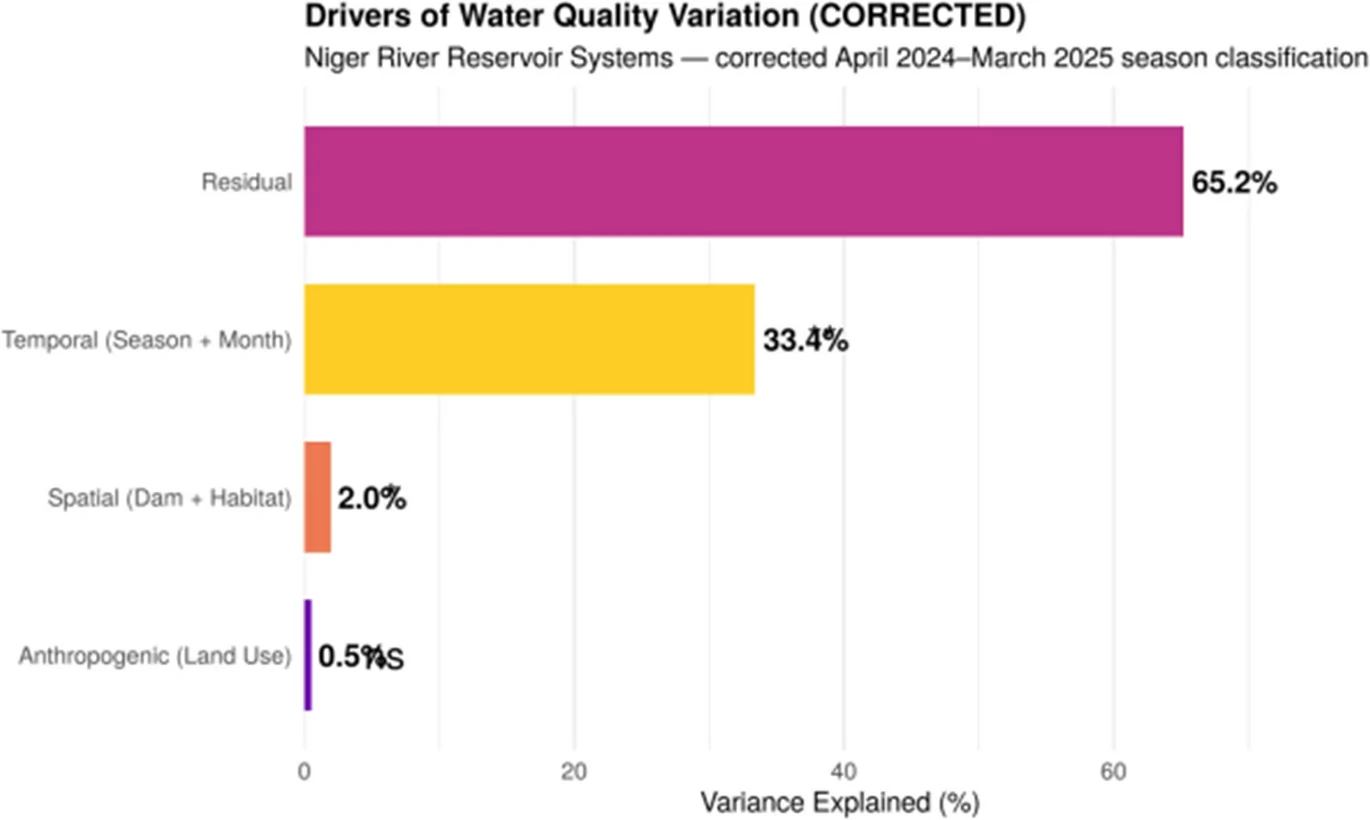

Fig. 6 Variance partitioning of water quality (adjusted R2 from conditional RDA, *n* = 72). Temporal factors dominate. ***p < 0.001; *p < 0.05; ns, not significant

In the Discussion section: Any quantitative references to the 28.3% temporal fraction should be updated to 33.4%.

The Results and Discussion paragraphs interpreting specific published Table 3 relationships, including relative humidity as a predictor of BOD at Kainji Lacustrine during the wet season, and air temperature as a predictor of pH and dissolved oxygen in lacustrine and ecotonal habitats during the wet season, describe relationships that do not appear in the corrected results and should be replaced with a discussion of the corrected Table 3 entries. Corrected Table 3 and Figures 5a–5b are given below.

**Table 3:** Near-significant Granger causality relationships from climate drivers to water-quality parameters across Jebba and Kainji, showing unadjusted and FDR-adjusted p-values

Site	Habitat	Season	Cause	Effect	Lag	P_Value	Adj_P	Notes	
Jebba	Riverine	Dry	Pressure	pH	1	0.005917	0.714697	Levels	
Jebba	Ecotonal	Dry	Pressure	BOD	1	0.006093	0.714697	Levels	
Jebba	Lacustrine	Dry	Pressure	PO4_P	1	0.010512	0.714697	Levels	
Jebba	Ecotonal	Wet	Rainfall	Turbidity	1	0.020064	0.714697	First-differenced	
Jebba	Ecotonal	Wet	Rainfall	pH	1	0.020339	0.714697	Levels	
Jebba	Lacustrine	Wet	Windspeed	pH	1	0.024994	0.714697	First-differenced	
Jebba	Lacustrine	Dry	Windspeed	DO	1	0.025185	0.714697	Levels	
Kainji	Riverine	Dry	Temp_Air	Turbidity	1	0.025611	0.714697	Levels	
Jebba	Ecotonal	Wet	Pressure	Turbidity	1	0.031036	0.714697	First-differenced	
Jebba	Lacustrine	Wet	RH	NO3_N	1	0.033638	0.714697	First-differenced	
Jebba	Lacustrine	Wet	Rainfall	Temp_Water	1	0.036304	0.714697	Levels	
Jebba	Ecotonal	Dry	Windspeed	BOD	1	0.041047	0.714697	Levels	
Jebba	Riverine	Dry	Windspeed	PO4_P	1	0.041653	0.714697	Levels	
Jebba	Riverine	Dry	Pressure	DO	1	0.044273	0.714697	Levels	
Jebba	Riverine	Dry	Pressure	Temp_Water	1	0.044273	0.714697	Levels	
Jebba	Ecotonal	Dry	Pressure	pH	1	0.044273	0.714697	Levels	
Jebba	Lacustrine	Dry	Pressure	pH	1	0.044273	0.714697	Levels	
Jebba	Lacustrine	Dry	Rainfall	DO	1	0.045117	0.714697	Levels	
Kainji	Lacustrine	Wet	Windspeed	Temp_Water	1	0.046926	0.714697	First-differenced	
Kainji	Riverine	Dry	RH	pH	1	0.047851	0.714697	Levels	
Kainji	Ecotonal	Dry	RH	DO	1	0.047851	0.714697	Levels	
Kainji	Ecotonal	Dry	RH	pH	1	0.047851	0.714697	Levels	
Kainji	Ecotonal	Dry	RH	Temp_Water	1	0.047851	0.714697	Levels	
Kainji	Lacustrine	Dry	RH	DO	1	0.047851	0.714697	Levels	
Kainji	Lacustrine	Dry	RH	Turbidity	1	0.047851	0.714697	Levels	
Kainji	Lacustrine	Dry	RH	Temp_Water	1	0.047851	0.714697	Levels	

**Figure:**
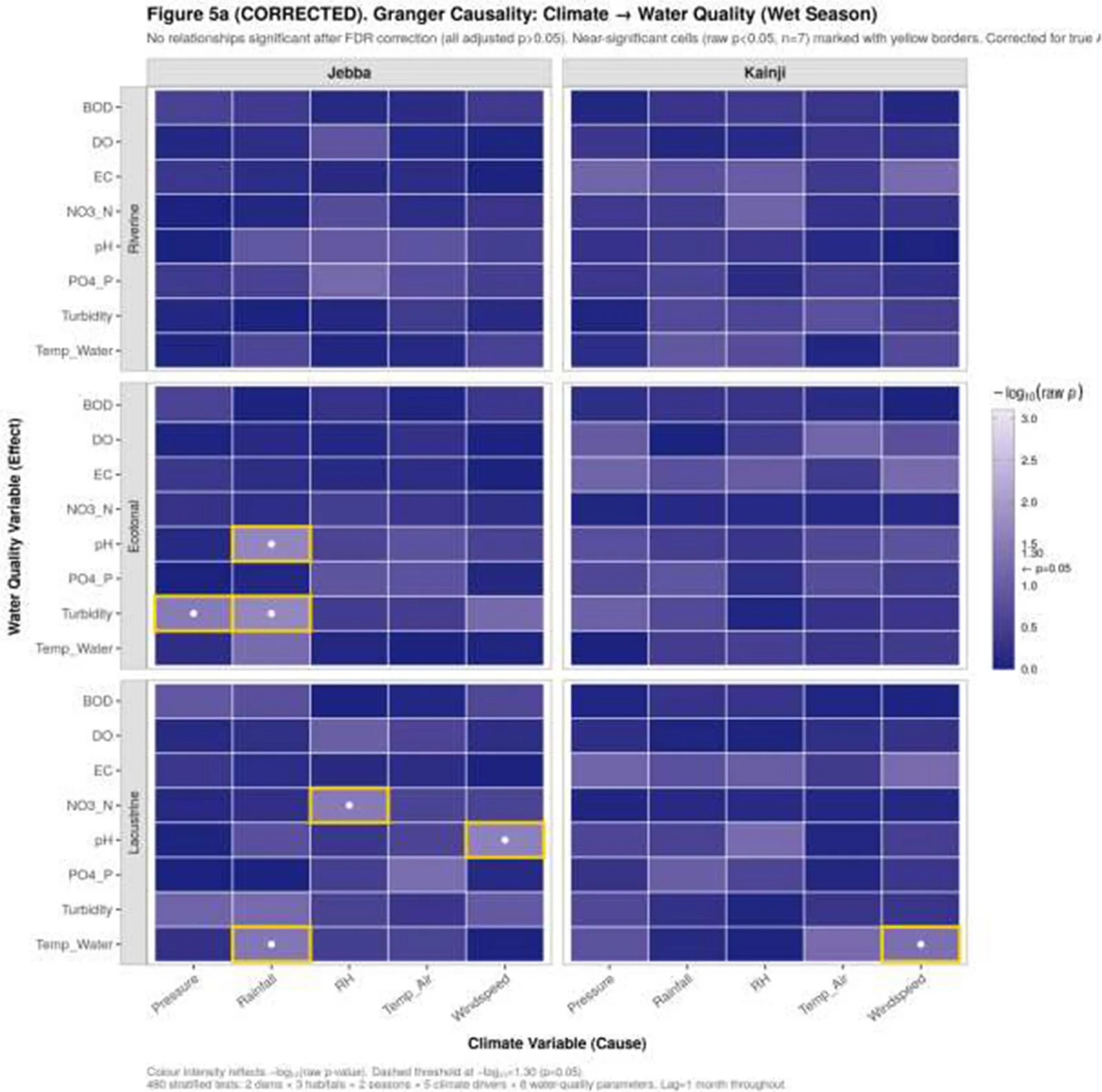

**Figure:**