# Supplementary material for: Temporal dynamics outweigh spatial gradients in shaping water quality across a regulated Afrotropical reservoir cascade
Source: Environ Monit Assess. 2026 Aug 3;198(9):906. doi: 10.1007/s10661-026-15681-8 (PMC13433401; doi:10.1007/s10661-026-15681-8)
Supplement: Supplementary file 1 [file 10661_2026_15681_MOESM1_ESM.docx]

**Supplementary Table Captions**

Table S1. Table S1. Full Granger causality analysis results underlying Figure 5a–b

Complete outputs of the exploratory Granger causality analysis testing monthly climate drivers against water‑quality parameters across the Kainji–Jebba reservoir cascade. The table reports all 480 stratified tests (2 dams × 3 habitat types × 2 seasons × 5 climate drivers × 8 water‑quality parameters) conducted with a fixed lag of 1 month. Raw and Benjamini–Hochberg false‑discovery‑rate (FDR)–adjusted p‑values are provided for each climate–water‑quality pair. Time‑series stationarity was assessed using the Augmented Dickey–Fuller test, with first‑differencing applied where required. Figure 5 is presented as two panels (wet season: Fig. 5a; dry season: Fig. 5b) solely for page‑layout and readability reasons; together, the panels summarise the full set of results reported in this table. No relationships remained statistically significant after FDR correction (all adjusted p > 0.05); results are therefore interpreted as exploratory and hypothesis‑generating given the one‑year dataset and short stratified time series

Supplementary Table S2. Statistical details for variance partitioning analysis. Corresponding data visualised in Figure 6.

**Supplementary Figure Captions**

**Supplementary Figure S1a**. Correlation matrix showing perfect agreement (r = 1.00) between WQI values calculated using Primary, Proportional, and Equal weighting schemes, confirming robustness of WQI calculations to parameter weight choices. Perfect correlations (r = 1.00) indicate that alternative weighting schemes alter magnitude but not relative WQI patterns.

**Supplementary Figure S1b**. Sensitivity analysis of variance partitioning to parameter weighting schemes. Venn diagrams show variance explained by spatial (X1), temporal (X2), anthropogenic (X3), and climate (X4) factors under Primary, Proportional, and Equal weighting schemes. Temporal factors (X2) consistently explain the most variance (~5.6-5.9%) across all schemes, confirming robustness of main findings.
