# Supplementary material for: Temporal dynamics outweigh spatial gradients in shaping water quality across a regulated Afrotropical reservoir cascade
Source: Environ Monit Assess. 2026 Aug 3;198(9):906. doi: 10.1007/s10661-026-15681-8 (PMC13433401; doi:10.1007/s10661-026-15681-8)
Supplement: Supplementary file 2 [file 10661_2026_15681_MOESM5_ESM.docx]

Supplementary Table S1. Statistical details for variance partitioning analysis. Corresponding data visualised in Figure 6.

| Component | Variance_pct | F_value | p_value | Significance |
| --- | --- | --- | --- | --- |
| Temporal (Season + Month) | 28.34159287 | 14.3212494 | 0.001 | ** |
| Spatial (Dam + Habitat) | 1.808804717 | 1.85018294 | 0.038 | * |
| Anthropogenic (Land Use) | 0.372622875 | 1.17514196 | 0.303 | ns |
| Residual | 70.20907252 | - | - |  |

Note: ** p < 0.01; * p < 0.05; ns = not significant
